# Cropland redistribution to marginal lands undermines environmental sustainability

**DOI:** 10.1093/nsr/nwab091

**Published:** 2021-05-22

**Authors:** Wenhui Kuang, Jiyuan Liu, Hanqin Tian, Hao Shi, Jinwei Dong, Changqing Song, Xiaoyong Li, Guoming Du, Yali Hou, Dengsheng Lu, Wenfeng Chi, Tao Pan, Shuwen Zhang, Rafiq Hamdi, Zherui Yin, Huimin Yan, Changzhen Yan, Shixin Wu, Rendong Li, Jiuchun Yang, Yinyin Dou, Wenbin Wu, Liqiao Liang, Bao Xiang, Shiqi Yang

**Affiliations:** Key Laboratory of Land Surface Pattern and Simulation, Institute of Geographic Sciences and Natural Resources Research, Chinese Academy of Sciences, Beijing 100101, China; Key Laboratory of Land Surface Pattern and Simulation, Institute of Geographic Sciences and Natural Resources Research, Chinese Academy of Sciences, Beijing 100101, China; International Center for Climate and Global Change Research, School of Forestry and Wildlife Sciences, Auburn University, Auburn, AL 36849, USA; International Center for Climate and Global Change Research, School of Forestry and Wildlife Sciences, Auburn University, Auburn, AL 36849, USA; Key Laboratory of Land Surface Pattern and Simulation, Institute of Geographic Sciences and Natural Resources Research, Chinese Academy of Sciences, Beijing 100101, China; Faculty of Geographical Science, Beijing Normal University, Beijing 100875, China; State Key Laboratory of Urban and Regional Ecology, Research Center for Eco-Environmental Sciences, Chinese Academy of Sciences, Beijing 100085, China; College of Resources and Environment, University of Chinese Academy of Sciences, Beijing 100049, China; School of Public Administration and Law, Northeast Agricultural University, Harbin 150030, China; Key Laboratory of Land Surface Pattern and Simulation, Institute of Geographic Sciences and Natural Resources Research, Chinese Academy of Sciences, Beijing 100101, China; College of Resources and Environment, University of Chinese Academy of Sciences, Beijing 100049, China; School of Geographical Sciences, Fujian Normal University, Fuzhou 350007, China; School of Resources and Environmental Economics, Inner Mongolia University of Finance and Economics, Hohhot 010017, China; School of Geography and Tourism, Qufu Normal University, Rizhao 276826, China; Northeast Institute of Geography and Agroecology, Chinese Academy of Sciences, Changchun 130012, China; Royal Meteorological Institute of Belgium, Brussels 1180, Belgium; Xinjiang Institute of Ecology and Geography, Chinese Academy of Sciences, Urumqi 830011, China; School of Geography and Tourism, Qufu Normal University, Rizhao 276826, China; Key Laboratory for Resources Use & Environmental Remediation, Institute of Geographic Sciences and Natural Resources Research, Chinese Academy of Sciences, Beijing 100101, China; Northwest Institute of Eco-Environment and Resources, Chinese Academy of Sciences, Lanzhou 730000, China; Xinjiang Institute of Ecology and Geography, Chinese Academy of Sciences, Urumqi 830011, China; Hubei Province's Key Laboratory for Environment & Disaster Monitoring and Evaluation, Innovation Academy for Precision Measurement Science and Technology, Chinese Academy of Sciences, Wuhan 430077, China; Northeast Institute of Geography and Agroecology, Chinese Academy of Sciences, Changchun 130012, China; Key Laboratory of Land Surface Pattern and Simulation, Institute of Geographic Sciences and Natural Resources Research, Chinese Academy of Sciences, Beijing 100101, China; Institute of Agricultural Resources and Regional Planning, Chinese Academy of Agricultural Sciences, Beijing 100081, China; Key Laboratory of Tibetan Environment Changes and Land Surface Processes, Institute of Tibetan Plateau Research, Chinese Academy of Sciences, Beijing 100101, China; Institute of Ecology, Chinese Research Academy of Environmental Sciences, Beijing 100012, China; College of Environment and Planning, Henan University, Kaifeng 475004, China

**Keywords:** cropland redistribution, environmental sustainability, marginal lands, wind erosion, irrigation water consumption

## Abstract

Cropland redistribution to marginal land has been reported worldwide; however, the resulting impacts on environmental sustainability have not been investigated sufficiently. Here we investigated the environmental impacts of cropland redistribution in China. As a result of urbanization-induced loss of high-quality croplands in south China (∼8.5 t ha^–1^), croplands expanded to marginal lands in northeast (∼4.5 t ha^–1^) and northwest China (∼2.9 t ha^–1^) during 1990–2015 to pursue food security. However, the reclamation in these low-yield and ecologically vulnerable zones considerably undermined local environmental sustainability, for example increasing wind erosion (+3.47%), irrigation water consumption (+34.42%), fertilizer use (+20.02%) and decreasing natural habitats (−3.11%). Forecasts show that further reclamation in marginal lands per current policies would exacerbate environmental costs by 2050. The future cropland security risk will be remarkably intensified because of the conflict between food production and environmental sustainability. Our research suggests that globally emerging reclamation of marginal lands should be restricted and crop yield boost should be encouraged for both food security and environmental benefits.

## Teaser

Widespread loss of high-quality cropland induced by urbanization and increasing shift of crop production to low-quality marginal land threaten food security and environmental sustainability.

## INTRODUCTION

The cultivated planet is withstanding record-breaking pressure to ensure food security. To meet the rising demand for food, energy and fiber, a 70–100% increase in crop commodities will be needed by 2050 [1–4]. However, food production is facing multiple challenges from urbanization, climate change and land degradation [5–8]. Urbanization alone, mainly in Asia and Africa, caused a 15.92 × 10^4^ km^2^ reduction of high-quality croplands during 1992–2016, which were 1.77 times more productive than the global average [5,9]. To boost crop production, two main pathways, cropland intensification and expansion, were adopted [2]. For example, at the global scale, land reclamation has been widely documented, particularly at the edges of the Amazon forest, Eurasian steppe and Sahara Desert, mostly from grassland and forest land [10]. Noticeably, the marginal lands have exhibited a large potential for increasing grain production as the pivotal mothball land resource [11,12]. The current estimate of marginal lands accounts for 36% of cultivated lands (1.3 × 10^9^ ha), which may provide food for 1/3 of the global population [12,13].

Marginal lands have intrinsically little potential value or profit in agricultural or industrial production because of their limited natural conditions and/or accessibility, that is poor soil, insufficient water supply, and prohibitive distances from roads and other means of transportation. Marginal lands are characteristically associated with low crop productivity, severe land degradation and high environmental risk [12,14]. Therefore, cropland redistribution to marginal lands has widely been concerned with optimizing land management and intensifying environmental protection [12]. In China, the marginal lands are becoming the pivotal pathway for supplementing the limited cultivated resource [15]. Here, *marginal land* is referred to as the new reclamation lands converted from grassland, wetland or other lands in the past 25 years, which are mostly distributed in northwest (NW), northeast (NE) and north (N) China.

**Figure 1.**
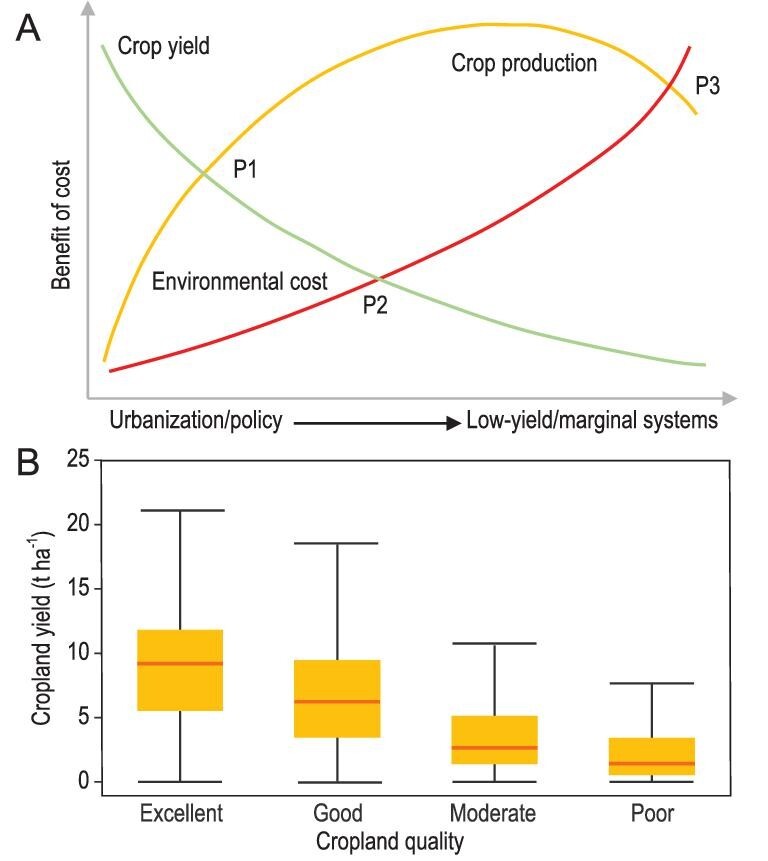
Framework for trade-off between food security and environmental costs as cropland redistributes to low-quality or marginal lands. (A) The impact of crop yield or production, and environmental costs resulting from crop redistribution to low-quality or marginal lands driven by urbanization and land management policies. (B) Large differences in crop yields in croplands of different quality levels in China.

Previous studies [16–18] show that high-quality croplands are usually related to high inputs like fertilizer and irrigation water. However, the environmental costs of cropland redistribution to low-quality or marginal land may be higher in the long term. These costs are multiple, such as land cost, water consumption, soil erosion and fertility decline. To describe the gain and loss of cropland redistribution, we developed a framework to represent the trade-off among crop yield, production and environmental cost (Fig. 1A). The principle of multiple disciplines from land economics, land resource and environmental sustainability was utilized as the theoretical basis in the current framework. Land competition is a core principle for varied human demands, that is settlement, food and ecology, because of the scarcity of the resource. According to the theory of agricultural location, croplands are replaced with settlements for housing with a high profit as the outcome of land competition. However, croplands with a relatively low profit were increasingly redistributed to marginal lands to ensure food security in certain areas or countries. From the perspective of potential productivity of radiation-temperature-water, those crops from the reclaimed lands might be subject to low yield or high environmental cost because of restrictive factors (Fig. 1B). Therefore, the consequence of land competition with the supplement of marginal lands may undermine national food security and environmental sustainability for the long term.

In this framework, three smooth curves are used to denote the rising environmental cost (red) and the decline in crop yield (green) in line with the increase of croplands on marginal lands (x axis). The gross crop production (yellow) shows an ascending trend. Correspondingly, environmental costs may also increase but at a relatively slow rate. At Point 1 (P1), crop production realizes growth at low environmental costs and decline of crop yield. At Point 2 (P2), crop yield further decreases but environmental costs start accelerating because of intensified land and management costs. When cropland expansion cannot compensate the crop yield loss, crop production will start to decrease. At Point 3 (P3), crop production will decrease rapidly as a result of exacerbated soil degradation, crop yield loss and cropland abandonment, and then show a decreasing trajectory induced by low yield and high environmental costs in specific regions (Fig. 1A). This means that high-quality croplands can have higher yields (e.g. 9.59 t ha^–1^ at Level ‘excellent’ and 7.11 t ha^–1^ at Level ‘good’ shown in Fig. 1B) with a lower environmental cost. The trade-off between grain production and environmental sustainability is pivotal at local, regional and global scales [19,20]. As a result of low per unit yield from marginal lands, which are mostly located in areas qualified as moderate or poor with average yields of 3.67 t ha^–1^ and 2.38 t ha^–1^, respectively (Fig. 1B), the reclaimed areas of marginal lands need to be expanded to offset crop production from high-quality cropland loss in specific areas. When the ascending proportion of marginal land reclamation in specific areas or countries exceeds a certain threshold for supplementing the crop production loss (Fig. 1A), the decline of crop yield slows down while the growth rate of environmental costs accelerates. Consequently, the proportion of marginal lands in specific areas or countries should be controlled through formal legislation to improve land policy and achieve environmental sustainability goals.

The environmental costs of reclamation of marginal land include different aspects, for example water resource protection, soil conservation, biodiversity and greenhouse gas emissions [21–24]. In this study, we focused on irrigation water consumption, soil erosion, fertilizer use and natural habitat loss (Supplementary Table S1).

China has a limited arable land resource and varied planting conditions, that is climatic, soil and water. It is challenging for China to feed more than 22% of the world's population with only 7% of the global croplands and 5% of globally utilizable freshwater [7,25]. In past decades, China has experienced rapid urbanization from 26.4% in 1990 [26] to 56.1% in 2015 (data available at http://www.stats.gov.cn/tjsj/ndsj/2015/indexeh.htm), with remarkable socioeconomic development (gross domestic product increased from $0.27 × 10^12^ to $9.75 × 10^12^). To protect cropland resources, national land-use policies have been successively promulgated [20,27], such as the policy for Cultivated Land Requisition-compensation Balance in 1998 (Supplementary Table S2). However, an unexpected outcome of these policies is the cropland redistribution to low-quality marginal lands [27,28], which caused multiple challenges from soil degradation, increase of irrigation water and overfertilization in China [20,25,29,30]. In light of the importance of pursuing food security, a comprehensive assessment of the impacts of these policies on China's cropland resources and environmental sustainability is essential. We use China as an example to validate our framework by assessing the associated food benefits and environmental costs from large-scale cropland redistribution driven by economic development and policy implementation.

## RESULTS

### Patterns of cropland redistribution to marginal lands across China

**Figure 2.**
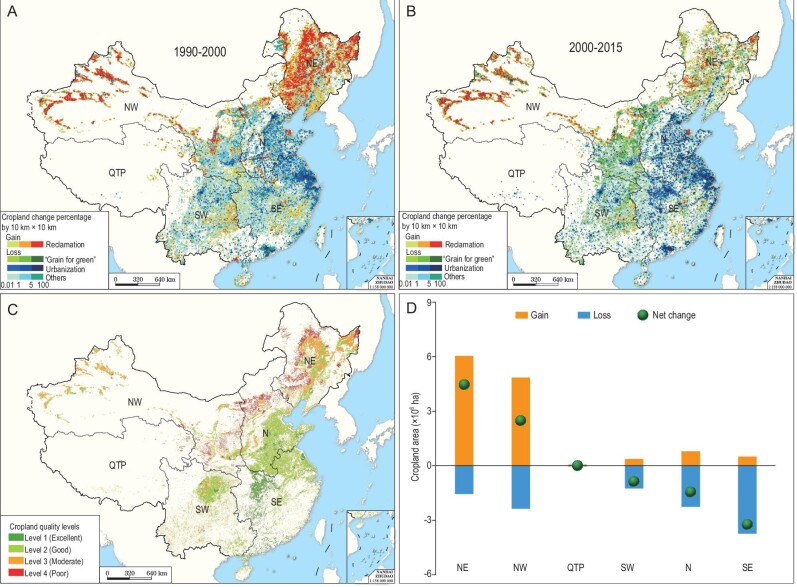
National cropland redistribution and change in cropland area in different zones, 1990–2015. (A) and (B) The spatial distributions of cropland gains and losses in 1990–2000 and 2000–2015. (C) The spatial distribution of croplands of different quality levels in 2015. (D) The net change of cropland area in different zones. NE, northeast China; NW, northwest China; N, north China; SE, southeast China; SW, southwest China; QTP, Qinghai-Tibet Plateau.

The results show that the cropland area in China was 138.04 × 10^6^ ha in 1990 and peaked in 2000 with a total area of 141.41 × 10^6^ ha (Fig. 2A and B; Supplementary Table S3). From 2000, the total cropland area experienced minor variations and was 139.47 × 10^6^ ha in 2015 (Supplementary Table S3). Underlying the relatively stable total area of croplands, however, are profound changes in spatial patterns of both cropland quantity and quality. During 1990–2015, 11.21 × 10^6^ ha of cropland was transformed, located mainly in southeast (SE), southwest (SW) and N China, while 12.64 × 10^6^ ha of cropland was reclaimed, mostly in NE and NW China (Supplementary Fig. S1 and Tables S3 and S4). A total of 79.1% cropland loss happened in the regions with high-quality croplands or with excellent farming conditions. In comparison, 73.6% of 12.64 × 10^6^ ha of newly reclaimed lands were moderate or poor cropland, as derived from the cropland quality investigation, mainly constrained by water shortages, soil degradation, topographic factors or fragile ecosystems (Fig. 2C). Conventional high-quality croplands, for example, those in the middle and lower Yangtze River plains and North China Plain (Fig. 2C; Supplementary Fig. S2), were encroached by urban and industrial expansion, resulting in decreases in cropland area by 3.24 × 10^6^ ha, 0.87 × 10^6^ ha and 1.46 × 10^6^ ha in SE, SW and N China, respectively (Fig. 2D; Supplementary Table S3). Thus only 82.7% of croplands which were present in 1990 are still preserved. Therefore, the cropland redistribution caused an increase of moderate and poor croplands from 42.3% to 48.9% across China (Supplementary Table S3).

NE China gained the largest increase of cropland area by 4.47 × 10^6^ ha during 1990–2015, mainly from grasslands (49.5%) and wetlands including swamp and water body (33.0%), of which 1.93 × 10^6^ ha were transformed to paddy fields (Fig. 2D; Supplementary Table S3). The concurrent total cropland area in NW China increased by 2.48 × 10^6^ ha, mainly through the reclamation of grasslands (74.9%) (Fig. 2D; Supplementary Table S3). Before 2000, reclamation in NE China dominated the increase in the national total cropland area. Since then, reclamation has occurred primarily in NW China, a large part of which traditionally had not been suitable for crop production because of the dry climate (Supplementary Fig. S1 and Table S3).

The new land reclamation in NW and NE China could be related to the policy of dynamic equilibrium of the total croplands in the country (Fig. 2D; Supplementary Table S2) and accordingly more benefits for agricultural companies, farmers and local governments, as well as exemption from agricultural tax since 2006. Note that there was considerable reclamation in oasis regions of Xinjiang before and after 2000 (Supplementary Fig. S1), where large areas of water-consuming cash crops (e.g. cotton and vegetables) are planted. The warming and wetting trends in the oasis areas [7] and recent improvements in groundwater irrigation technologies provide more suitable farming conditions than ever. Rapid demand for food through the increase in the local population has also facilitated the rapid expansion of large-scale oases in arid regions since 2000 [31–33]. Although ecological restoration projects converted an extra 2.34 × 10^6^ ha of croplands to forests or grasslands, mainly distributed in NW, SW and N China during 2000–2015 (Fig. 2B; Supplementary Fig. S1), the overall quality of croplands indicated a general decreasing trend across China in the last 25 years.

### Potential food production reduction because of cropland redistribution

**Figure 3.**
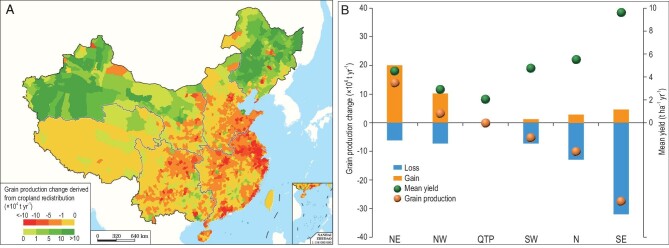
Impact of the national cropland redistribution on grain production, 1990–2015. (A) Spatial distribution of grain production change caused by cropland redistribution at county level. (B) Net changes, gains and losses of grain production caused by cropland redistribution, and the mean yield of cropland in each zone.

The cropland redistribution yielded an increase in grain production in NE and NW China but a decrease in SE, SW and N China (Fig. 3A; Supplementary Table S5). In SE and SW China, which have relatively high yield (on average 8.53 t ha^–1^ yr^–1^) and two or three crop rotations (Supplementary Figs S3 and S4), the cropland loss of 3.74 × 10^6^ ha and 1.25 × 10^6^ ha, respectively, resulted in a decrease in grain production by 31.92 × 10^6^ t yr^–1^ and 6.77 × 10^6^ t yr^–1^, respectively (Fig. 2; Supplementary Fig. S3 and Tables S3 and S5). In N China, the cropland loss of 2.26 × 10^6^ ha reduced grain production by 12.96 × 10^6^ t yr^–1^, while NW and NE China have relatively low yields (on average 2.89 t ha^–1^ yr^–1^ and 4.50 t ha^–1^ yr^–1^, respectively) and one crop rotation, the reclaimed cropland of 10.89 × 10^6^ ha led to an increase in crop production by 10.42 × 10^6^ t yr^–1^ and 20.11 × 10^6^ t yr^–1^, respectively (Fig. 3B; Supplementary Tables S3 and S5). However, the production increase in these regions could not compensate for the grain production losses in SW, SE and N China (Supplementary Table S5).

On average, production of 1 ton of grain needs only 0.10 ha, 0.19 ha or 0.18 ha of croplands in SE, SW or N China, respectively, whereas 0.35 ha in NW China or 0.22 ha in NE China is needed to produce the same amount. Consequently, at the country level, the crop redistribution contributed to 4.5% reduction in grain production (equivalent to feeding 57 × 10^6^ people), although the total cropland area remained steady under the supervision of various aforementioned policies [28] (Supplementary Table S2). We also examined China's grain imports from other countries during the study period, which persistently escalated from 13.72 × 10^6^ t yr^–1^ in 1990 to 124.77 × 10^6^ t yr^–1^ in 2015 to provide enough food for the country. Meanwhile, the grain import dependency ratio rose substantially from 2.98% to 16.72% (http://www.stats.gov.cn/english/Statisticaldata/AnnualData/). Therefore, the cropland redistribution also brought an increase in food insecurity in China.

### Environmental costs of cropland redistribution

According to the trade-off theory between food security and environmental costs, the increase of cropland area by relatively high-quality land reclamation can enhance grain productivity and therefore improve food supply with low environmental cost in the initial stage, which is represented by P1 in Fig. 1A. Because of the limited land resource in a given country or region, a large amount of low-quality or marginal lands will be reclaimed accompanied by increasing environmental costs and declining crop yield. Their equilibrium point is situated at P2 in Fig. 1A.

**Figure 4.**
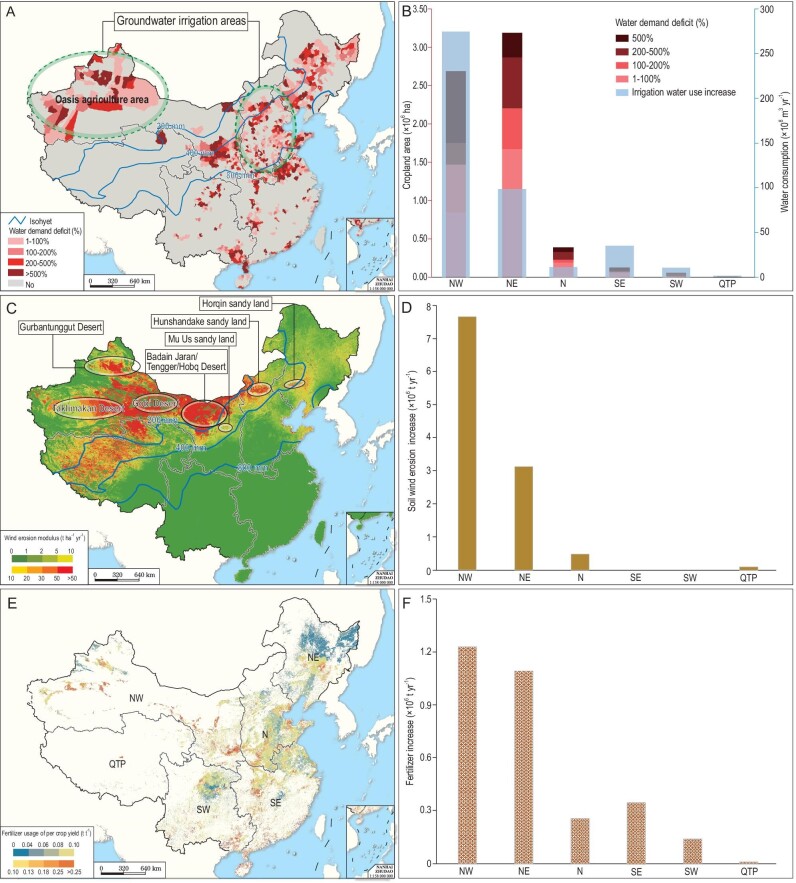
Environmental effects of national cropland redistribution on irrigation water use and soil erosion by wind. (A) Spatial distribution of water demand deficit. (B) Cropland area and proportion of different water demand deficit levels, and the irrigation water use increase resulting from cropland gain in each zone. (C) Spatial distribution of average wind erosion modules. (D) Effect of cropland gain on soil erosion by wind in each zone. (E) Spatial distribution of average fertilizer use per crop yield. (F) The increase of fertilizer use resulting from cropland gain in each zone.

Besides crop production loss, environmental ramifications induced by cropland redistribution are also emerging, particularly in the ecologically fragile zones of NW China [32]. Based on the above theory, land degradation was exacerbated by agriculture-induced water resource scarcity and intensified wind erosion in line with large-scale land reclamation. The croplands (4.85 × 10^6^ ha) converted from grasslands or oases in NW China are primarily located in arid and semi-arid areas with mean annual precipitation less than 200 mm yr^–1^ (Fig. 4A; Supplementary Table S4). The precipitation can meet only 18.7% to ∼54.0% of crop water demand in NW China, resulting in an increase in irrigation water use by 278.65 × 10^8^ m^3^ yr^–1^ (37.92% of irrigation water use in 2015) because of cropland expansion during 1990–2015 (Fig. 4B; Supplementary Table S6). As more than half of the croplands in NW China (55.4%) were located in water demand deficit areas after redistribution, extracting groundwater for irrigation caused continuous groundwater depletion [33] (Fig. 4A and B; Supplementary Table S6 and Fig. S5). For example, a sharp drop of the groundwater table at a rate of 4.2 m decade^–1^ has been reported in Minqin oasis [34]. Cropland expansion in grasslands also has seriously aggravated soil erosion by wind, increasing the erosion modulus from 5.68 t ha^–1^ yr^–1^ to 29.79 t ha^–1^ yr^–1^ (Fig. 4C and D; Supplementary Table S7 and Fig. S6). Notably, the total wind erosion from croplands around the Gobi Desert and sand areas increased by 7.64 × 10^6^ t yr^–1^, which has seriously offset the effectiveness of the ecological restoration projects such as ‘Grain for green’ since 2000 [35,36] (Fig. 4C; Supplementary Table S7). The efficiency of fertilizer use in NW China is the lowest among all zones, representing a maximum fertilizer use (0.15 t) to produce 1 ton of crop production across China. As a result, the land reclamation has caused an extra increase of 1.22 × 10^6^ t fertilizer use in this zone (Fig. 4E and F; Supplementary Table S8 and Fig. S7).

In NE China, dryland reclamation and paddy fields expansion from 1990 to 2015 increased the volume of irrigation water use by 100.40 × 10^8^ m^3^ yr^–1^, because approximately 53% of the reclaimed cropland was located in water demand deficit areas (Fig. 4A and B; Supplementary Table S6) while local precipitation (∼800 mm yr^–1^) meets only ∼60.3% of crop water demand in paddy fields (Fig. 4A). The reclamation in NE China also exacerbated soil organic matter loss, especially in black soil areas, which severely accelerated land degradation [37]. Despite the high efficiency of fertilizer use in NE China, the massive land reclamation resulted in an increasing amount of fertilizer use to 1.10 × 10^6^ t yr^–1^ (Fig. 4E and F; Supplementary Table S8). Additionally, crop expansion encroaches natural habitats of various plants and animals, undermining ecosystem services, and environmental sustainability [2,38].

In summary, the croplands in NE and NW China are characterized with relatively low quality and low productivity, and 80.2% of land reclamation originated on moderate and poor lands. The grain production of 1 ton from reclaimed lands caused approximately an extra 1.69 × 10^3^ m^3^ yr^–1^ in irrigation water consumption (compared to the national average of 1.12 × 10^3^ m^3^ yr^–1^), 0.73 t yr^–1^ in soil erosion from wind (national average: 0.11 t yr^–1^) and 0.15 t yr^–1^ in fertilizer use (national average: 0.10 t yr^–1^) in NW China, which shows the highest environmental costs among all zones. As a result, land reclamation in NE China and NW China created the 10.75 × 10^6^ t yr^–1^ increase in wind erosion, which accounts for 3.47% across all croplands; it also increased the consumption of irrigation water by 379.05 × 10^8^ m^3^ yr^–1^ and fertilizer use by 2.32 × 10^6^ t yr^–1^, which contributed to 34.42%, and 20.02% increases across all croplands, respectively (Supplementary Tables S6, S7 and S8). The environmental costs in water use and black soil degradation were also high in NE China. We computed the 62.58% of reclamation which was converted from swamps and grasslands in NE China, and the massive grasslands (74.90%) which were reclaimed as ecological barriers in semi-arid and arid NW China. Our investigation estimated that 3.11% of natural habitat disappeared between 1990 and 2015 because of cropland reclamation, which will accelerate biodiversity loss in those fragile ecosystem zones [23,39]. Therefore, the cropland redistribution to marginal lands has diminished ecosystem services and caused environmental deterioration in both NE and NW China, which has transgressed the situation at P2 in Fig. 1A, creating an unsustainable status of food security and environment.

### Impacts of cropland redistribution on environmental sustainability in the future

**Figure 5.**
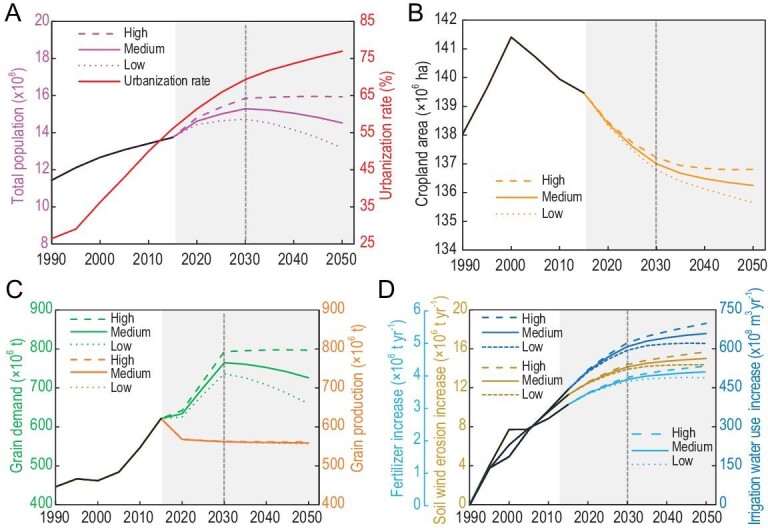
The predicted total population, urbanization rate, cropland area, grain demand and supply, and environmental costs of cropland reclamation under high, medium and low scenarios from 2020 to 2050 in China. (A) The urbanization rate will ascend continuously from 2020 to 2050, and the predicted total population will reach a peak in 2030. (B) The total cropland area will successively decrease because of future rapid urbanization and reach the lowest level in 2050. (C) The national cereal demand will first increase until 2030 and then decrease in 2030–2050, which will result in serious cereal supply and demand contradictions and will reach the maximum for national food insecurity in 2030. (D) If cropland redistribution patterns continue under the condition of unimproved cropland management, environmental sustainability will be persistently undermined in view of the massive increases in wind erosion, and irrigation water and fertilizer uses.

We also projected crop redistribution by 2050 under various population and urbanization scenarios with fixed cropland protection policies. The results showed that, in a medium scenario, China's population will reach 1.53 × 10^9^ in 2030 and decrease to 1.45 × 10^9^ in 2050, with corresponding grain demand at 764.50 × 10^6^ t and 726.14 × 10^6^ t, respectively (Fig. 5A; Supplementary Tables S9 and S10). Compared to 2015, an additional 0.34 × 10^9^ people will move to cities by 2050, resulting in further cropland loss of 3.22 × 10^6^ ha, mostly from high-quality farmland (Fig. 5B; Supplementary Table S9 and Fig. S8). Consequently, the projected national food demand will surpass the current annual grain production (572.28 × 10^6^ t) by 33.59% in 2030 (Fig. 5C; Supplementary Table S10).

According to our estimation, if the trajectory of cropland redistribution continues in the future, the quantity of croplands will successively decrease to 135.66–136.81 × 10^6^ ha in 2050. The high-quality cropland areas will continue to be encroached by urbanization based on the spatially explicated map of cropland change prediction (Supplementary Fig. S8). As a result, those high-yield croplands will be occupied by swelling cities across the nation, replaced by marginal lands that will not be able to meet the rising food demand. Land degradation will accelerate in the following decades (Fig. 2D), the irrigation water use will increase by 177.68–254.68 × 10^8^ m^3^ yr^–1^, soil erosion by wind will increase by 3.08–4.41 × 10^6^ t yr^–1^ and fertilizer use on reclaimed lands will increase by 0.83–1.19 × 10^6^ t yr^–1^ between 2015 and 2050 (Fig. 5D; Supplementary Table S10). According to the trade-off framework, the divergence between crop production and environmental cost will become larger in the future, driven by marginal land expansion, which is confirmed by the projections (Fig. 5C and D; Supplementary Table S10). Therefore, projected future cropland redistribution will severely exacerbate the conflicts between human beings and the environment and potentially undermine environmental sustainability.

## DISCUSSION

These environmental costs, either emerging or potential, from cropland redistribution to marginal lands in China are a straightforward reflection of a series of land-management policies [20,28], revealing that consideration of food-environment tensions is warranted in policy decisions. The purpose of these cropland protection policies is to ensure food security without dampening economic development. From this view, these policies succeeded in guaranteeing ∼140 × 10^6^ ha of croplands for food demand [27,39]. However, cropland redistribution associated with these policies has resulted in high environmental costs [20]. According to a novel assessment, global urban expansion has been accelerating since the beginning of the twenty-first century [40]. Because of high proximity between high-quality farmland and cities, global urban expansion mainly encroaches the croplands with high productivity [5,41]. To fill the food gap, the large-scale reclamation happened in some marginal lands in NE and NW China, and other areas in the world such as the edge of the Amazon forest, Eurasian Steppe and Sahara Desert [10,27]. Therefore, cropland redistribution to marginal lands is becoming a global phenomenon. This study indicates that cropland redistribution is not sustainable for either food security or environmental sustainability in the long term. According to the trade-off theory, other developing countries, especially those in Africa and Asia, could experience similar trajectories of cropland changes and the accompanied environmental issues (Fig. 1). From this perspective, China's land management policies can give a profound lesson and enlightenments for these countries in combating hunger and malnutrition [20,41].

The prediction indicated that global urbanization will continue to drive cropland redistribution in the future [5]. Global urban expansion will encroach 1.8–2.4% of high-yield croplands with a decrease of 3–4% of crop production by 2030, and 80% of the cropland loss will happen in Asia and Africa [5]. As a result, the urbanization-induced cropland redistribution will continue globally into the next decade. Therefore, large-scale cropland redistribution could globally aggravate the spatial mismatch between the food demand and crop production, and cause more severe starvation. The world's undernourished and hungry population live in Asia and Sub-Saharan Africa with more than 3.81 × 10^8^ and 2.50 × 10^8^, respectively. By 2030, the number of people affected by hunger would surpass 8.40 × 10^8^ [42]. Because of the low potential of crop production in those countries, a large amount of land reclamation will happen in marginal lands, including hotspot areas of biodiversity, soil degradation and water scarcity [6,16,24,43]. With a higher soil erosion modulus in croplands of those countries, land degradation will be enhanced in those areas with land reclamation [36], which will especially aggravate desertification in some semi-arid and arid areas in the future. We also found that the efficiency of fertilizer and irrigation water uses is especially low in arid areas, and cropland reclamation will accelerate land degradation, including groundwater depletion, land salinization and desertification. Meanwhile, we found that land reclamation induced a 2.32 × 10^6^ t yr^–1^ increase in fertilizer use in NE and NW China (Supplementary Table S8). With concurrent water resource scarcity, the pollution of ponds, lakes and rivers is aggravated by increased fertilizer use in agriculture, which certainly will undermine the quality of the water environment [44]. This process of cropland redistribution to marginal lands implicates a subsequent shift in amounts of fertile soil, irrigation water and fertilizer use in the vulnerable areas of agricultural production. Thus, global grain production will stagnate or decease while devastating environmental consequences will be increasingly serious in the future, threatening the livelihoods of local people [22]. We also conclude that the global equilibrium point may move to P3 in Fig.1A in the future, in which global food safety and environmental sustainability will be at high risk [16,45]. Consequently, cropland redistribution to marginal lands may undermine global environmental sustainability [22].

To address those issues, we thus appeal for improvements in current cropland management policies to control both cropland loss and reclamation by taking the environmental ramifications into account. First, the existing high-quality croplands should be given a priority to prevent the encroachment from urbanization [20,46]. Second, attention should be given to avoid future land reclamation from ecologically fragile hotspots [47,48]. Third, water-saving measures, such as using sprinkler and drip irrigation systems [23,24,49], allowing cropland fallow, and cropping rotations in marginal lands should be adopted to enhance environmental sustainability [20].

However, cropland redistribution under global warming will positively impact agricultural production. For example, the increase of available water resources including warming-induced glacier melt and water diversion from inland rivers would be especially important in improving the water scarcity for crops in NW China in the future. Some arid and semi-arid regions might become wetter, while drier conditions are projected for other areas. Therefore, the negative impacts of cropland redistribution to marginal lands on environmental sustainability might be mitigated under wetter conditions.

Our simulation indicated that China's grain provision will not meet the country's food demand by 2050 at the current agriculture technical level. However, technological improvements will positively enhance food provision for the future growing population. For example, the integrated soil-crop system management practices increase average yields for rice, wheat and maize, and might produce more grain with lower environmental costs [50]. Also, the continuous increase of import and export trade accompanying economic development will amplify food provision for direct human consumption and animal feed in the future.

The land governance strategies for the areas of large-scale cropland redistribution should pay more attention to the potential effects of marginal land reclamation. Scientific and rational policies should be addressed to safeguard the potential land-use shift to achieve the win-win goals of food security and environmental sustainability under the 2030 United Nations (UN) Sustainable Development Goals (SDGs) [51].

## METHODS

### Agro–Big data in China

We developed spatially explicit datasets including cropland dynamics, grain production, irrigation water use, wind erosion and fertilizer use with pixel and county scales across China (Supplementary Figs S1, S3, S5, S6 and S7). The datasets on national cropland quality levels and cropping systems were also collected (Supplementary Figs S2 and S4). We also acquired 1629 questionnaires and surveyed 236 sample plots of cropland (Supplementary Table S1 and Fig. S9).

### Cropland dynamic analysis

Cropland is defined as cultivated land for producing grain and vegetables, including paddyland with irrigation facilities and dry farmland for three-year-planted crops [27] (Supplementary Table S11). The national cropland datasets with 30-m resolution and vector dynamic patches at 5-year intervals from 1990 to 2015 were extracted from the 1 : 100 000 China Land Use/cover Dataset (CLUD) based on Landsat TM/ETM+, HJ-1A/1B, and Landsat 8 OLI images (Supplementary Fig. S1). These datasets were generated using a uniform classification method with artificially visual interpretation aided by geo-knowledge [27,52–55]. Here a systematic sampling scheme was developed to calculate the net area of cropland by considering the proportion of small non-cropland objects from mosaicked farmland in different zones, which may efficaciously remove unidentified features in remotely sensed images, that is field roads, canals, ditch facilities for irrigation and dispersed settlements [27] (Supplementary Fig. S10). The overall accuracy of first-level land types was 91.95% in 2015. The accuracies of cropland classification were more than 90.36% from 1990 to 2015 [27,28,52–55] (Supplementary Table S12). We also compiled the implemented policies for land use and cropland protection in this period (Supplementary Table S2).

### The overall impacts of cropland changes

Here we quantified the contributions of cropland gain (reclamation) and loss (encroachment) on staple crops production under present technological levels. The average yield per unit of cropland as a baseline in 2011–2015 was accurately retrieved to eliminate the uncertainties associated with interannual climate variation or statistical data.

We calculated the net area of cropland gain or loss of each county multiplied by its average yield (Supplementary Table S3) using GIS-based spatial analysis. The contribution of cropland gain or loss on grain production at national scale is computed as follows:

$$\begin
{equation*}\quad\quad{P_z} = \sum\limits_{i = 1}^n {{A_i} \times {Y_i}},
\end{equation*}$$

where ${P_z}$ is the total grain production gain or loss across the country; *i* and *n* are the *i*^th^ county and total number of counties, respectively; ${A_i}$ is the area of cropland change in *i*^th^ county in a specific period; ${Y_i}$ is the average yield per unit in this period, which was calculated using total grain production and net cropland area of the *i*^th^ county.

To quantify and evaluate the impact of cropland reclamation on regional irrigation water, two key indexes were used: irrigation water use and water demand deficit for crops. The available water resource for crop planting was calculated by starting with the total water capacity at county level in China and subtracting water use for industries, residents and ecology as detailed in the *China Water Resources Bulletin* of the Ministry of Water Resources (Supplementary Fig. S5). Then the irrigation water use (Supplementary Fig. S5) was retrieved by multiplying the area of newly reclaimed cropland, percentage of irrigated area and water consumption per unit area:

$$\begin
{equation*}{W_z} = \sum\limits_{i = 1}^n {{A_i} \times {I_i} \times {C_{i,irrigation}}} ,\end{equation*}$$

where ${W_z}$ is irrigation water use for a specific zone; ${A_i}$ is the area of cropland gain in *i*^th^ county in a specific period; ${I_i}$ is the percentage of irrigation area occupying total cropland area; ${C_{i, irrigation}}$ is water consumption per unit area for irrigation, which is calculated as the average level in 2011–2015. The estimated irrigation water use was validated using the field investigation from the State Agriculture Comprehensive Development Office (Supplementary Fig. S11).

Thus, we define the water demand deficit for crop planting as the difference between actual irrigation water use and total available water volume for planting crops (Supplementary Fig. S5) in a specific zone, expressed as:

$$\begin
{equation*}{\rm{\ }}{D_z} = \frac{{{W_z} - {W_a}}}{{{W_a}}}{\rm{\ }} \times {\rm{\ }}100{\rm{\% }},\end{equation*}$$

where ${D_z}$ is the water demand deficit ratio for crops and ${W_a}$ is the available water volume for crops.

Based on our previous studies, we parameterized the revised wind erosion equation (RWEQ) and calibrated the model [56]. The spatially explicit dataset of average wind erosion modulus with 1 km × 1 km resolution was retrieved for 1991–2015 (Supplementary Fig. S6) and validated using ^137^Cs measures [57]. We calculated increases of wind erosion triggered by cropland redistribution in each county as follows:

$$\begin
{equation*}{\rm{\ }}{{\rm{S}}_z} = \mathop \sum \limits_{i = 1}^n {A_i} \times ({R_{i, after}} - {R_{i, before}}),\end{equation*}$$

where ${{\rm{S}}_z}$ is the net change in total soil wind erosion in a specific zone; *i* and *n* are the *i*^th^ county and total number of counties within a specific zone, respectively; ${A_i}$ is the area of cropland change in *i*^th^ county in a specific period; ${R_{i, after}}$ and ${R_{i, before}}$ are each county's average wind erosion modulus after and before cropland shifts, respectively, from 1991 to 2015.

We acquired the fertilizer use per cropland area of each county in 2011–2015 (Supplementary Fig. S7). Then we calculated the average fertilizer use per yield in this period as the fertilizer use efficiency in present conditions. The fertilizer use increase resulting from cropland reclamation in the period of 1991–2015 was assessed at the county scale through spatial analysis. We calculated the increases of fertilizer use caused by cropland reclamation (gain) in each zone as follows:

$$\begin
{equation*}\quad\quad{\rm{\ }}{T_{\ z}} = \mathop \sum \limits_{i = 1}^n {A_i}\ \times \ {F_i},\end{equation*}$$

where ${T_z}$ is the increase in fertilizer use in a specific zone; *i* and *n* are the *i*^th^ county and total number of counties within a specific zone, respectively; ${A_i}$ is the area of cropland gain in *i*^th^ county in a specific period; ${F_i}$ is each county's average fertilizer use per cropland area in present conditions.

### Projection of future cropland change and its impacts on food security and environment

We projected the future food demand driven by population growth, a new wave of urbanization and improved livelihood towards no hunger, food security and sustainable cities, which are closely related to the UN SDGs [42]. The UN adopted the high, medium and low variants of world population prospects to project the total population of each country [58]. We forecast the total population from 2020 to 2050 based on the growth rate of population, incorporating China's two-child policy [59] (Supplementary Table S9):

$$\begin
{equation*}P\!o\!{p_f}\! \left( t \right) = P\!o\!{p_{f, un}}\! \left( t \right)\! +\! {\Delta _{2016,t}}\left( C \right),\end{equation*}$$

where $P\!o\!{p_f}( t )$ is the predicted total population under high, medium and low scenarios in future *t* period;${\rm{\ }}P\!o\!{p_{f, un}}\! ( t )$ is the UN’s predicted population with high, medium and low scenarios; ${\Delta _{2016, t}}( C )\ $is the estimated increase in population since the implementation of China's two-child policy.

Future national grain demand was estimated based on the projected total population and per capita use of calories and protein with a baseline in 2015 under high, medium and low scenarios (Supplementary Table S10). In the high, medium or low scenarios with a 2015 baseline of 400 kg, the grain demand will reach 450 kg per capita by 2030 [29,58]:

$$\begin
{equation*}F\!o\!o{d_f}\! \left( t \right) = \ P\!o\!{p_f}\!\left( t \right)\ \times \ DF\!\! \left( t \right),\end{equation*}$$

where $F\!o\!o\!{d_f}( t )$ is the predicted grain demand under high, medium and low scenarios in future *t* period; $DF\! ( t )$ is the grain demand per capita in a different future period.

We estimated the future urbanization rate of China based on the UN’s projection of world urbanization prospects in 2017 [58]. The future urban land demand was projected based on a logistic regression equation between urban area and urban population for 1990–2015. In parallel, spatially explicit probability was mapped based on Purdue University's Land Transformation Model (http://ltm.agriculture.purdue.edu/ltm/default.htm) [60].

$$\begin
{eqnarray*}
&&A{L_{f, crop \leftrightarrow others}}\!\! \left( t \right)\nonumber\\
&&\quad = P ( {A_{urban}}\!( {P\!o\!{p_f}\!\left( t \right)\!,{R_{urbanization}}\!\!\left( t \right)\ }\!),\\
&&\quad\quad L\!\! \left( {{D_c},{D_{rail}},{D_h},{D_{ri}},{D_s}, E, S} \right) ),\end{eqnarray*}$$

where $A{L_{f, crop \leftrightarrow others}}\!( t )$ is the predicted shift between cropland and other land types under high, medium and low scenarios in future *t* period; ${A_{urban}}( {P\!o\!{p_f}( t ),\ {R_{urbanization}}( t )})$ is the predicted occupation resulting from urban expansion, which is related to predicted total population $( {P\!o\!{p_f}( t )} )$ and the predicted urbanization rate (${R_{urbanization}}( t )$); $L\! ( {{D_c},\ {D_{rail}},\ {D_h},\ {D_{ri}},\ {D_s},\ E,\ S} )$ is the spatial weight coefficient related to distance from city center, railway, highway, river, shoreline, relative elevation and slope factors, which were calculated through neural network learning [60]. Finally, we depicted spatially explicit probability maps of future urban expansion with 1 km × 1 km spatial resolution under high, medium and low scenarios in 2030 and 2050 (Supplementary Fig. S8).

We also assessed the effects of future cropland change in the current redistribution pattern on grain production, irrigation water use, soil erosion from wind and fertilizer use across the country from 2020 to 2030 and 2050 (Supplementary Table S10). The spatially statistical analysis was adopted to assess these effects using the aforementioned formulas in a GIS environment.

$$\begin
{equation*}{P_f}(t) = \sum\limits_{j = 1}^m {{A_j}(t) \times {Y_j}},
\end{equation*}$$

$$\begin
{equation*}{W_f}(t) = \sum\limits_{j = 1}^m {{A_j}(t) \times {I_j} \times {C_{j,irrigation}},} \end{equation*}$$

$$\begin
{equation*}{{\rm{S}}_{f }}\!\left( {{t}} \right){\rm{\ }} = \mathop \sum \limits_{j = 1}^m {A_j}\!\left( t \right)\ \times ({R_{j, after}}\ - \ {R_{j, before}}),\end{equation*}$$

$$\begin
{equation*} {T_f}\!\left( {{t}} \right){\rm{\ }} = \mathop \sum \limits_{j = 1}^m {A_{j }}\!\left( t \right)\ \times {F_j}\ ,\end{equation*}$$

where ${P_f}( t )$, ${W_f}( t )$, ${S_{f }}( t )$, ${T_f}( t )$ are the changes in grain production, irrigation water use, soil erosion from wind, and fertilizer use, respectively, under high, medium and low scenarios from 1990 to future *t* period. The *j* and *m* are the *j*^th^ zone and total number of zones, respectively. ${A_j}( t )$ is the total changing area of cropland gain in *j*^th^ zone from 2015 to *t* period. ${Y_j}$, ${I_j},\ {C_{j, irrigation}}$ are the average yield per unit, percentage of irrigation area occupying the total cropland area and water use per unit area for irrigation, respectively. ${R_{j, before}}$, ${R_{j, after}}$ are each county's average wind erosion modulus before and after cropland shifts, and ${F_j}$ is the average fertilizer use per cropland area in *j*^th^ zone.

### Sources of uncertainty and quality control

Series of quality-control measures were conducted in the status analysis and future projections. We analyzed national cropland area changes using vector dynamic patches to ensure statistical accuracy with consistent time-series datasets available from CLUD. The net cropland area was obtained by eliminating unidentified objects from remotely sensed images with less than two pixels (or 60-m resolution). We validated the net cropland area of 980 random samples with a 1 km × 1 km grid across China in 2015 (Supplementary Table S12.2 and Fig. S10). The grain productivity was assessed by cross-validation with remotely sensed data, statistical cereal yield per unit by county, and inventories of farmers and managers (Supplementary Fig. S10). The 1629 questionnaires from agricultural management were applied to validate irrigation water and fertilizer uses (Supplementary Fig. S9). The spatially explicit dataset on the wind erosion modulus was validated using ^137^Cs measures. Future projections of population, urbanization rate, urban expansion and grain demand indexes were estimated based on UN and FAO estimates and taking into account China's macropolicies.

## DATA AVAILABILITY

All datasets used in this study are available to the public or included in the Supplementary data. In addition, the datasets that support the findings of this study are available upon reasonable request to W.K. (kuangwh@igsnrr.ac.cn).

## Supplementary Material

nwab091_Supplemental_File

## References

[1] Godfray HCJ , Beddington JR, Crute IR et al. Food security: the challenge of feeding 9 billion people. Science 2010; 327: 812–8. 10.1126/science.1185383 20110467

[2] Zabel F , Delzeit R, Schneider JM et al. Global impacts of future cropland expansion and intensification on agricultural markets and biodiversity. Nat Commun 2019; 10: 2844. 10.1038/s41467-019-10775-z 31253787 PMC6598988

[3] Kastner T , Rivas MJI, Koch W et al. Global changes in diets and the consequences for land requirements for food. Proc Natl Acad Sci USA 2012; 109: 6868–72. 10.1073/pnas.1117054109 22509032 PMC3345016

[4] Tilman D , Balzer C, Hill J et al. Global food demand and the sustainable intensification of agriculture. Proc Natl Acad Sci USA 2011; 108: 20260–4. 10.1073/pnas.1116437108 22106295 PMC3250154

[5] Bren d’Amour C , Reitsma F, Baiocchi G et al. Future urban land expansion and implications for global croplands. Proc Natl Acad Sci USA 2017; 114: 8939–44. 10.1073/pnas.1606036114 28028219 PMC5576776

[6] Montgomery DR. Soil erosion and agricultural sustainability. Proc Natl Acad Sci USA 2007; 104: 13268–72. 10.1073/pnas.0611508104 17686990 PMC1948917

[7] Piao SL , Ciais P, Huang Y et al. The impacts of climate change on water resources and agriculture in China. Nature 2010; 467: 43–51. 10.1038/nature09364 20811450

[8] Tian HQ , Ren W, Tao B et al. Climate extremes and ozone pollution: a growing threat to China's food security. Ecosyst Health Sustain 2016; 2: e01203. 10.1002/ehs2.1203

[9] Huang QX , Liu ZW, He CY et al. The occupation of cropland by global urban expansion from 1992 to 2016 and its implications. Environ Res Lett 2020; 15: 084037. 10.1088/1748-9326/ab858c

[10] Tan MH , Li YY. Spatial and temporal variation of cropland at the global level from 1992 to 2015. J Resour Ecol 2019; 10: 235–45.

[11] Brown LR. World population growth, soil erosion, and food security. Science 1981; 214: 995–1002. 10.1126/science.7302578 7302578

[12] Kang S , Post WM, Nichols JA et al. Marginal lands: concept, assessment and management. J Agric Sci 2013; 5: 129–39.

[13] Wood S , Sebastian K, Scherr SJ. Pilot Analysis of Global Ecosystems: Agroecosystems. Washington, DC: International Food Policy Research Institute and World Resources Institute, 2000.

[14] Barbier EB. Sustaining agriculture on marginal land. Environment 1989; 31: 12–40.

[15] Liu JY , Zhang ZX, Xu XL et al. Spatial patterns and driving forces of land use change in China during the early 21st century. J Geogr Sci 2010; 20: 483–94. 10.1007/s11442-010-0483-4

[16] Folberth C , Khabarov N, Balkovič J et al. The global cropland-sparing potential of high-yield farming. Nat Sustain 2020; 3: 281–9. 10.1038/s41893-020-0505-x

[17] Matson PA , Parton WJ, Power AG et al. Agricultural intensification and ecosystem properties. Science 1997; 277: 504–9. 10.1126/science.277.5325.504 20662149

[18] Poore J , Nemecek T. Reducing food's environmental impacts through producers and consumers. Science 2018; 360: 987–92. 10.1126/science.aaq0216 29853680

[19] Balmford A , Amano T, Bartlett H et al. The environmental costs and benefits of high-yield farming. Nat Sustain 2018; 1: 477–85. 10.1038/s41893-018-0138-5 30450426 PMC6237269

[20] Kong XB. China must protect high-quality arable land. Nature 2014; 506: 7. 10.1038/506007a 24499883

[21] Foley JA , DeFries R, Asner GP et al. Global consequences of land use. Science 2005; 309: 570–4. 10.1126/science.1111772 16040698

[22] Tilman D , Fargione J, Wolff B et al. Forecasting agriculturally driven global environmental change. Science 2001; 292: 281–4. 10.1126/science.1057544 11303102

[23] Phalan B , Onial M, Balmford AR et al. Reconciling food production and biodiversity conservation: land sharing and land sparing compared. Science 2011; 333: 1289–91. 10.1126/science.1208742 21885781

[24] Flörke M , Schneider C, McDonald RI. Water competition between cities and agriculture driven by climate change and urban growth. Nat Sustain 2018; 1: 51–8. 10.1038/s41893-017-0006-8

[25] Zuo L , Zhang ZX, Carlson KM et al. Progress towards sustainable intensification in China challenged by land-use change. Nat Sustain 2018; 1: 304–13. 10.1038/s41893-018-0076-2

[26] National Bureau of Statistics of the People's Republic of China . China Statistical Yearbook. Beijing: China Statistics Press, 1991.

[27] Liu JY , Liu ML, Tian HQ et al. Spatial and temporal patterns of China's cropland during 1990–2000: an analysis based on landsat TM data. Remote Sens Environ 2005; 98: 442–56. 10.1016/j.rse.2005.08.012

[28] Kuang WH , Liu JY, Dong JW et al. The rapid and massive urban and industrial land expansions in China between 1990 and 2010: a CLUD-based analysis of their trajectories, patterns, and drivers. Landsc Urban Plan 2016; 145: 21–33. 10.1016/j.landurbplan.2015.10.001

[29] Liu XB , Zhang XY, Herbert SJ. Feeding China's growing needs for grain. Nature 2010; 465: 420. 10.1038/465420a 20505710

[30] Lu YL , Jenkins A, Ferrier RC et al. Addressing China's grand challenge of achieving food security while ensuring environmental sustainability. Sci Adv 2015; 1:e1400039. 10.1126/sciadv.1400039 26601127 PMC4644077

[31] Chen C , Park T, Wang XH et al. China and India lead in greening of the world through land-use management. Nat Sustain 2019; 2: 122–9. 10.1038/s41893-019-0220-7 30778399 PMC6376198

[32] Du TS , Kang SZ, Zhang XY et al. China's food security is threatened by the unsustainable use of water resources in north and northwest China. Food Energy Secur 2014; 3: 7–18. 10.1002/fes3.40

[33] Liu MH , Jiang Y, Xu X et al. Long-term groundwater dynamics affected by intense agricultural activities in oasis areas of arid inland river basins, northwest China. Agric Water Manage 2018; 203: 37–52. 10.1016/j.agwat.2018.02.028

[34] Sun Y , Kang SZ, Li FS et al. Comparison of interpolation methods for depth to groundwater and its temporal and spatial variations in the Minqin oasis of northwest China. Environ Modell Softw 2009; 24: 1163–70. 10.1016/j.envsoft.2009.03.009

[35] Ouyang ZY , Zheng H, Xiao Y et al. Improvements in ecosystem services from investments in natural capital. Science 2016; 352: 1455–9. 10.1126/science.aaf2295 27313045

[36] Borrelli P , Bobinson DA, Fleischer LR et al. An assessment of the global impact of 21^st^ century land use change on soil erosion. Nat Commun 2017; 8: 2013. 10.1038/s41467-017-02142-7 29222506 PMC5722879

[37] Zhao PZ , Li S, Wang EH et al. Tillage erosion and its effect on spatial variations of soil organic carbon in the black soil region of China. Soil Tillage Res 2018; 178: 72–81. 10.1016/j.still.2017.12.022

[38] Verburg PH , Crossman N, Ellis EC et al. Land system science and sustainable development of the earth system: a global land project perspective. Anthropocene 2015; 12: 29–41. 10.1016/j.ancene.2015.09.004

[39] Kuang WH. 70 years of urban expansion across China: trajectory, pattern, and national policies. Sci Bull 2020; 65: 1970–4. 10.1016/j.scib.2020.07.005 36659053

[40] Kuang WH , Du GM, Lu DS et al. Global observation of urban expansion and land-cover dynamics using satellite big-data. Sci Bull 2021; 66: 297–300. 10.1016/j.scib.2020.10.022 36654403

[41] Gu BJ , Zhang XL, Bai XM et al. Four steps to food security for swelling cities. Nature 2019; 566: 31–3. 10.1038/d41586-019-00407-3 30718889

[42] Sustainable Development Solutions Network . Getting Started with the Sustainable Development Goals. https://sustainabledevelopment.un.org/content/documents/2217Getting%20started.pdf (3 June 2021, date last accessed).

[43] Jägermeyr J , Pastor A, Biemans H et al. Reconciling irrigated food production with environmental flows for sustainable development goals implementation. Nat Commun 2017; 8: 15900. 10.1038/ncomms15900 28722026 PMC5524928

[44] Lu CQ , Tian HQ. Global nitrogen and phosphorus fertilizer use for agriculture production in the past half century: shifted hot spots and nutrient imbalance. Earth Syst Sci Data 2017; 9: 181–92. 10.5194/essd-9-181-2017

[45] Ray DK , Ramankutty N, Mueller ND et al. Recent patterns of crop yield growth and stagnation. Nat Commun 2012; 3: 1293. 10.1038/ncomms2296 23250423

[46] Seto KC , Reenberg A, Boone CG et al. Urban land teleconnections and sustainability. Proc Natl Acad Sci USA 2012; 109: 7687–92. 10.1073/pnas.1117622109 22550174 PMC3356653

[47] Gao L , Bryan BA. Finding pathways to national-scale land-sector sustainability. Nature 2017; 544: 217–22. 10.1038/nature21694 28406202

[48] Foley JA , Ramankutty N, Brauman KA et al. Solutions for a cultivated planet. Nature 2011; 478: 337–42. 10.1038/nature10452 21993620

[49] Zhou F , Bo Y, Ciais P et al. Deceleration of China's human water use and its key drivers. Proc Natl Acad Sci USA 2020; 117: 7702–11. 10.1073/pnas.1909902117 32209665 PMC7148580

[50] Chen XP , Cui ZL, Fan MS et al. Producing more grain with lower environmental costs. Nature 2014; 514: 486–9. 10.1038/nature13609 25186728

[51] Liu JG , Hull V, Godfray HCJ et al. Nexus approaches to global sustainable development. Nat Sustain 2018; 1: 466–76. 10.1038/s41893-018-0135-8

[52] Liu JY , Zhang ZX, Zhuang DF et al. A study on the spatial-temporal dynamic changes of land-use and driving forces analyses of China in the 1990s. Geogr Res 2003; 22: 1–12.

[53] Liu JY , Zhang ZX, Zhuang DF et al. Remote Sensing Information Study of Land Use Change in China in 1990s. Beijing: Science Press, 2005.

[54] Ning J , Liu JY, Kuang WH et al. Spatiotemporal patterns and characteristics of land-use change in China during 2010–2015. J Geogr Sci 2018; 28: 547–62. 10.1007/s11442-018-1490-0

[55] Zhang ZX , Wang X, Zhao XL et al. A 2010 update of national land use/cover database of China at 1 : 100000 scale using medium spatial resolution satellite images. Remote Sens Environ 2014; 149: 142–54. 10.1016/j.rse.2014.04.004

[56] Chi WF , Zhao YY, Kuang WH et al. Impacts of anthropogenic land use/cover changes on soil wind erosion in China. Sci Total Environ 2019; 668: 204–15. 10.1016/j.scitotenv.2019.03.015 30852197

[57] Hu YF , Liu JY, Zhuang DF et al. Distribution characteristics of ^137^Cs in wind-eroded soil profile and its use in estimating wind erosion modulus. Chin Sci Bull 2005; 50: 1155–9. 10.1360/04wd0312

[58] United Nations (UN) . World Population Prospects: The 2017 revision population database. https://esa.un.org/unpd/wpp/DataQuery/ (3 June 2021, date last accessed).

[59] Wang KY , Li YX, Ding J. The influence of Chinese population policy change on resources and the environment. Chin J Popul Resour Environ 2016; 14: 227–34. 10.1080/10042857.2016.1258797

[60] Pijanowski BC , Brown DG, Shellito BA et al. Using neural networks and GIS to forecast land use changes: a land transformation model. Comput Environ Urban 2002; 26: 553–75. 10.1016/S0198-9715(01)00015-1

