# Supplementary material for: Cropland redistribution to marginal lands undermines environmental sustainability
Source: Natl Sci Rev. 2021 May 22;9(1):nwab091. doi: 10.1093/nsr/nwab091 (PMC8776548; doi:10.1093/nsr/nwab091)
Supplement: nwab091_Supplemental_File [file nwab091_supplemental_file.docx]

**Supplementary Data for**

**C****ropland redistribution to marginal lands undermines environmental sustainability**

Wenhui Kuang^1^ , Jiyuan Liu^1*^, Hanqin Tian^2*^, Hao Shi^2^, Jinwei Dong^1^, Changqing Song^3^, Xiaoyong Li^4,5^, Guoming Du^6^, Yali Hou^1,5^, Dengsheng Lu^7^, Wenfeng Chi^8^, Tao Pan^9^, Shuwen Zhang^10^, Rafiq Hamdi^11,14^, Zherui Yin^9^, Huimin Yan^12^, Changzhen Yan^13^, Shixin Wu^14^, Rendong Li^15^, Jiuchun Yang^10^, Yinyin Dou^1^, Wenbin Wu^16^, Liqiao Liang^17^, Bao Xiang^18^ and Shiqi Yang^19^

^1^ Key Laboratory of Land Surface Pattern and Simulation, Institute of Geographic Sciences and Natural Resources Research, Chinese Academy of Sciences, Beijing 100101, China

^2^ International Center for Climate and Global Change Research, School of Forestry and Wildlife Sciences, Auburn University, Auburn, AL 36849, USA

^3^ Faculty of Geographical Science, Beijing Normal University, Beijing 100875, China

^4^ State Key Laboratory of Urban and Regional Ecology, Research Center for Eco-Environmental Sciences, Chinese Academy of Sciences, Beijing 100085, China

^5^ College of Resources and Environment, University of Chinese Academy of Sciences, Beijing 100049, China

^6^ School of Public Administration and Law, Northeast Agricultural University, Harbin 150030, China

^7^ School of Geographical Sciences, Fujian Normal University, Fuzhou 350007, China

^8^ School of Resources and Environmental Economics, Inner Mongolia University of Finance and Economics, Hohhot 010017, China

^9^ School of Geography and Tourism, Qufu Normal University, Rizhao 276826, China

^10^ Northeast Institute of Geography and Agroecology, Chinese Academy of Sciences, Changchun 130012, China

^11^ Royal Meteorological Institute of Belgium, Brussels 1180, Belgium

^12^ Key Laboratory for Resources Use & Environmental Remediation, Institute of Geographic Sciences and Natural Resources Research, Chinese Academy of Sciences, Beijing 100101, China

^13^ Northwest Institute of Eco-Environment and Resources, Chinese Academy of Sciences, Lanzhou 730000, China

^14^ Xinjiang Institute of Ecology and Geography, Chinese Academy of Sciences, Urumqi 830011, China

^15^ Hubei Province's Key Laboratory for Environment & Disaster Monitoring and Evaluation, Innovation Academy for Precision Measurement Science and Technology, Chinese Academy of Sciences, Wuhan 430077, China

^16^ Institute of Agricultural Resources and Regional Planning, Chinese Academy of Agricultural Sciences, Beijing 100081, China

^17^ Key Laboratory of Tibetan Environment Changes and Land Surface Processes, Institute of Tibetan Plateau Research, Chinese Academy of Sciences, Beijing 100101, China

^18^ Institute of Ecology, Chinese Research Academy of Environmental Sciences, Beijing 100012, China

^19^ College of Environment and Planning, Henan University, Kaifeng 475004, China

* Corresponding authors. E-mails: [liujy@igsnrr.ac.cn](mailto:liujy@igsnrr.ac.cn); [tianhan@auburn.edu](mailto:tianhan@auburn.edu)

**Supplementary Figures S1*–*S11**

**Supplementary Tables S1*–*S12**

**
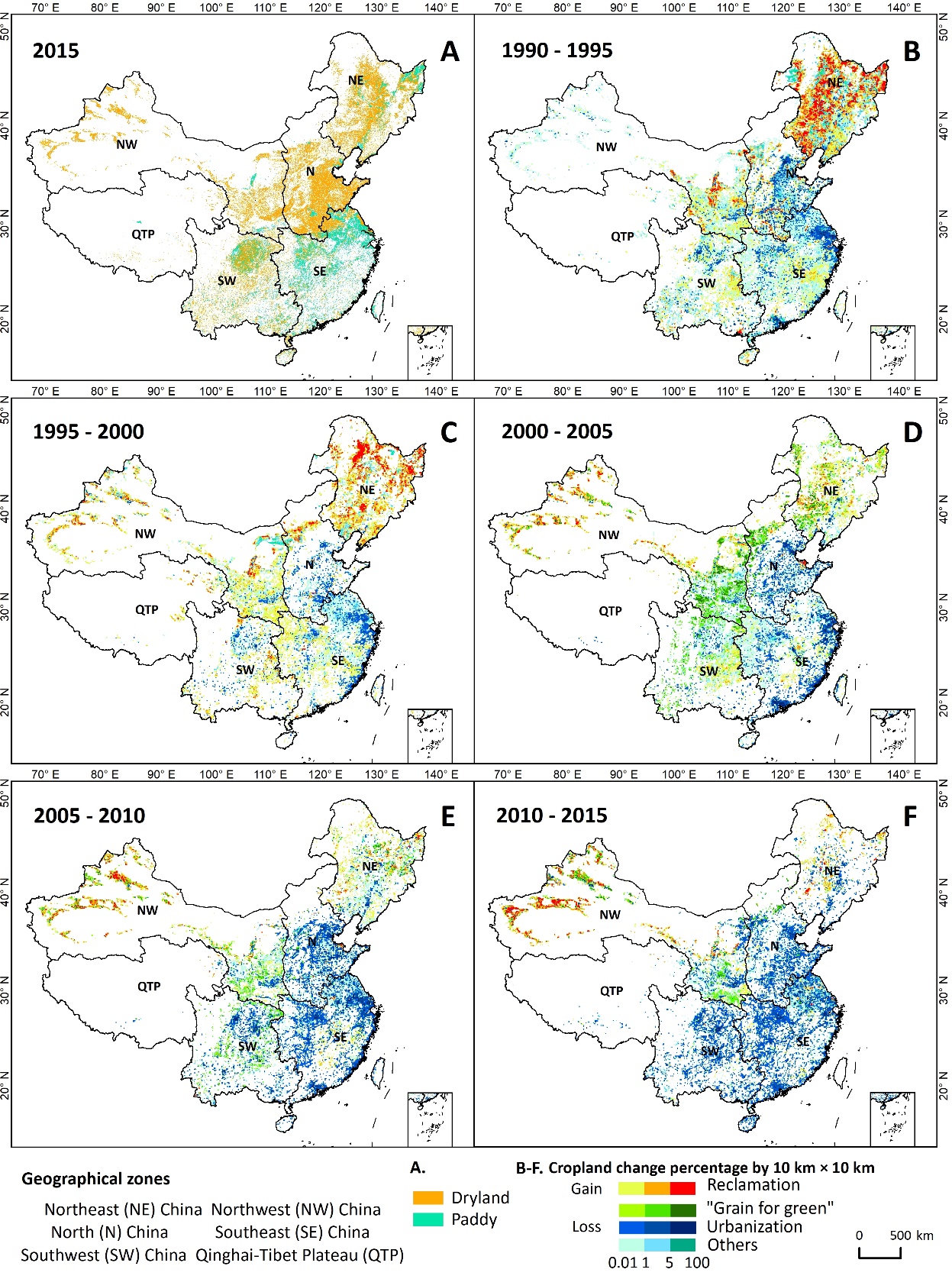
**

**Figure S1. National cropland distribution in 2015 and comparison of spatial distributions of cropland changes at five-year intervals between 1990 and 2015.** (A) Cropland distribution in 2015. (B-F) Cropland changes at five-year intervals.


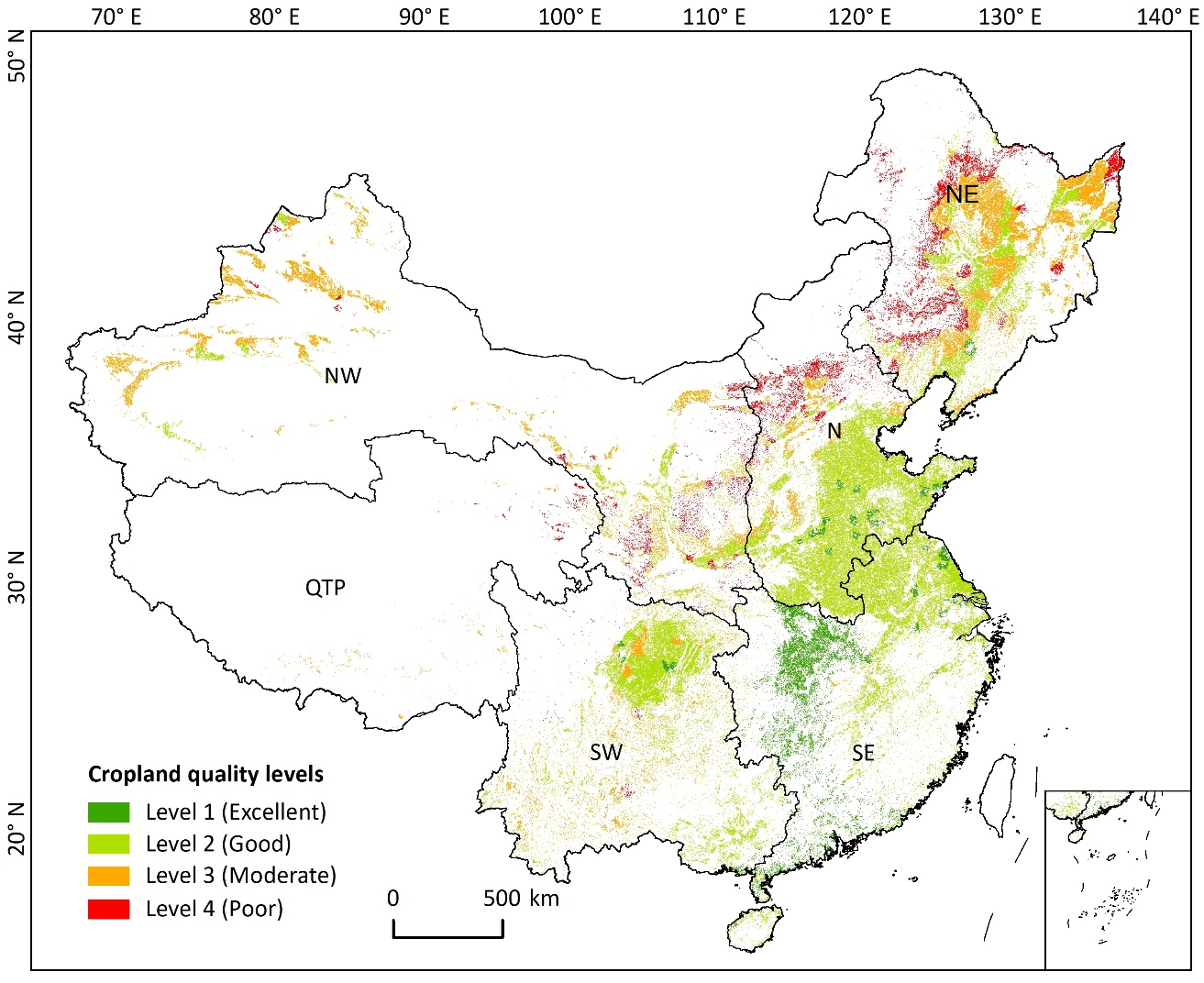


**Figure S2. Spatial distribution of cropland quality levels in China.** Note: NE, northeast China; NW, northwest China; N, north China; SE, southeast China; SW, southwest China; QTP, Qinghai-Tibet Plateau.

**
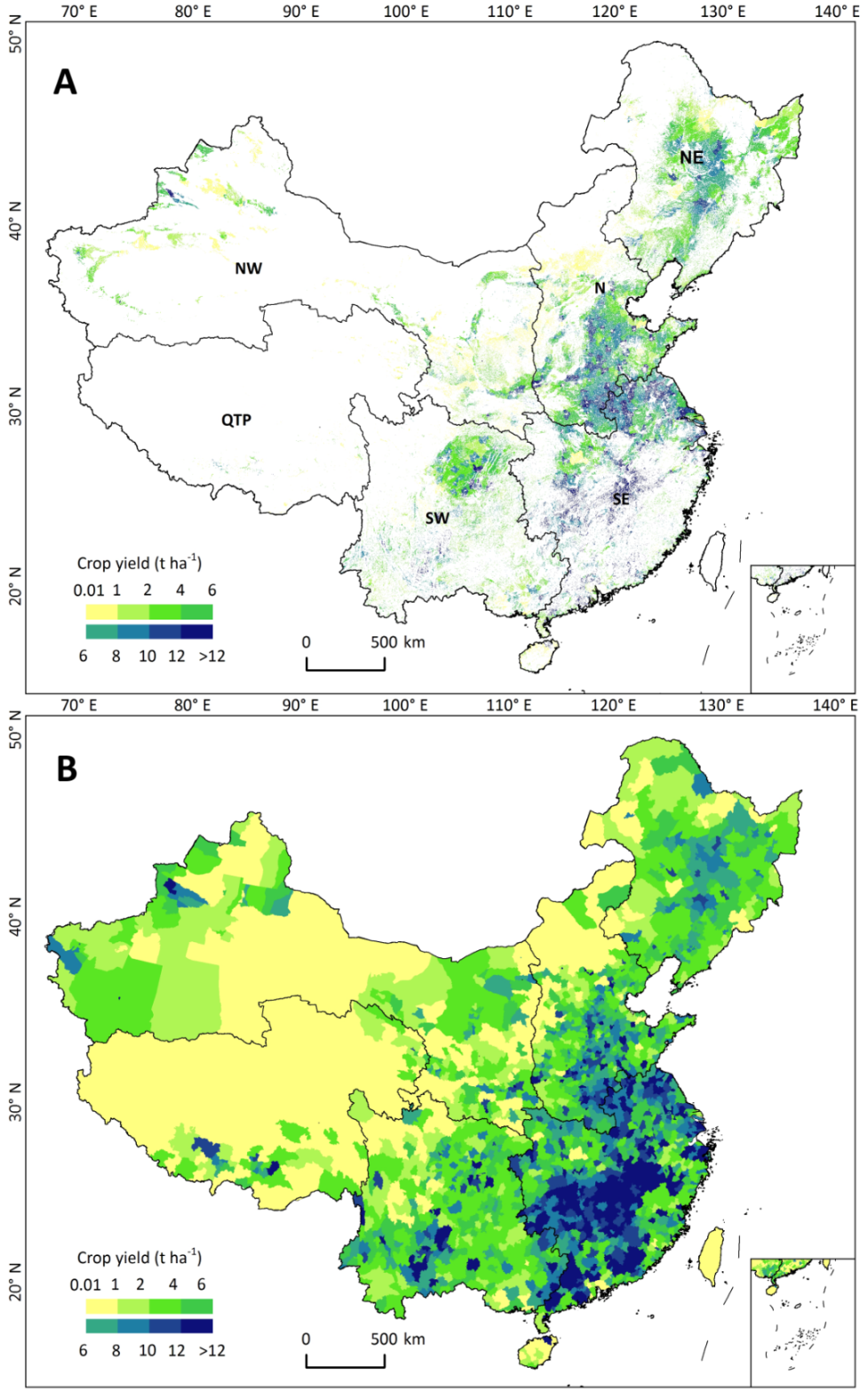
**

**Figure S3. Average grain productivity per unit at grid level (1 km×1 km) (A) and at county level (B).** Note: NE, northeast China; NW, northwest China; N, north China; SE, southeast China; SW, southwest China; QTP, Qinghai-Tibet Plateau.

**
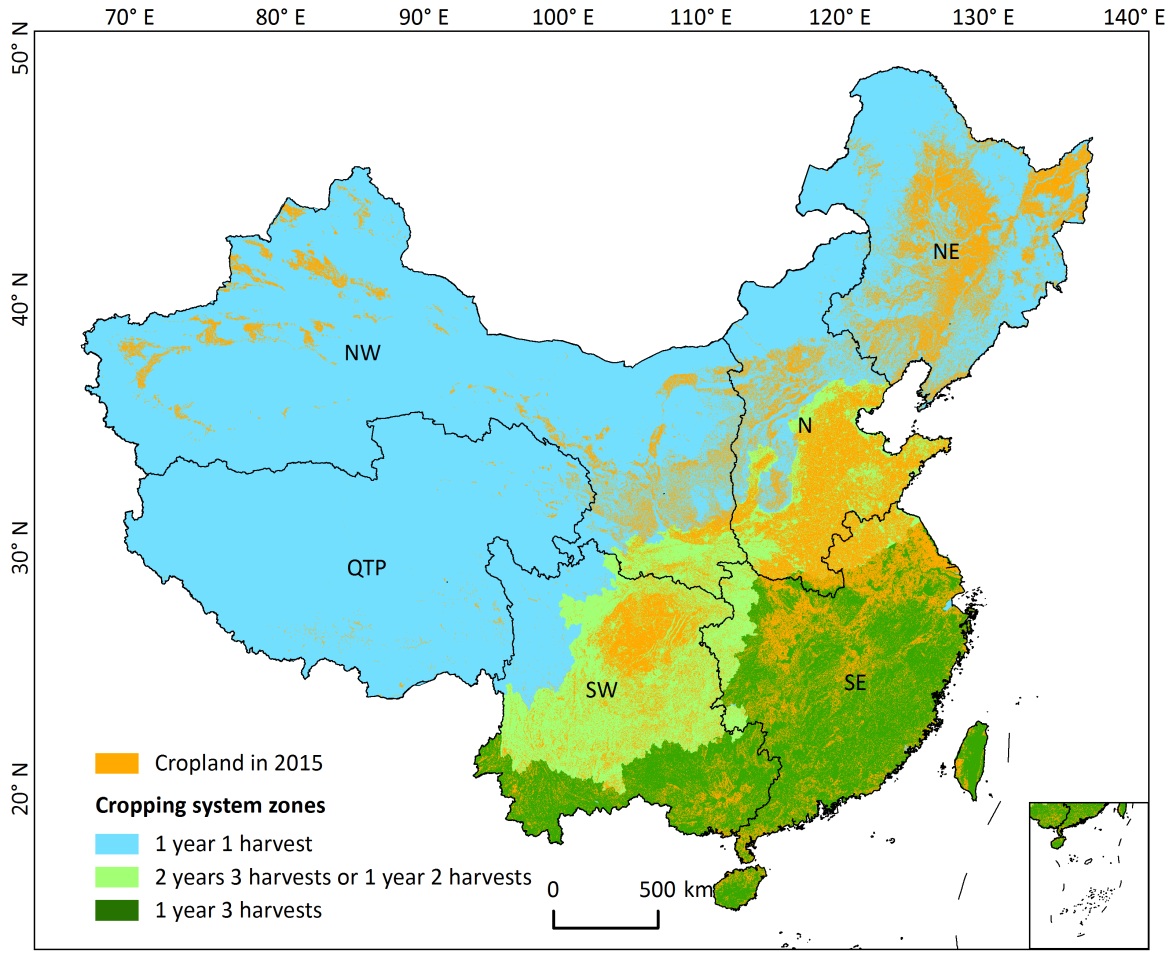
**

**Figure S4. Map of cropping systems used in China, 2015.** Note: NE, northeast China; NW, northwest China; N, north China; SE, southeast China; SW, southwest China; QTP, Qinghai-Tibet Plateau.


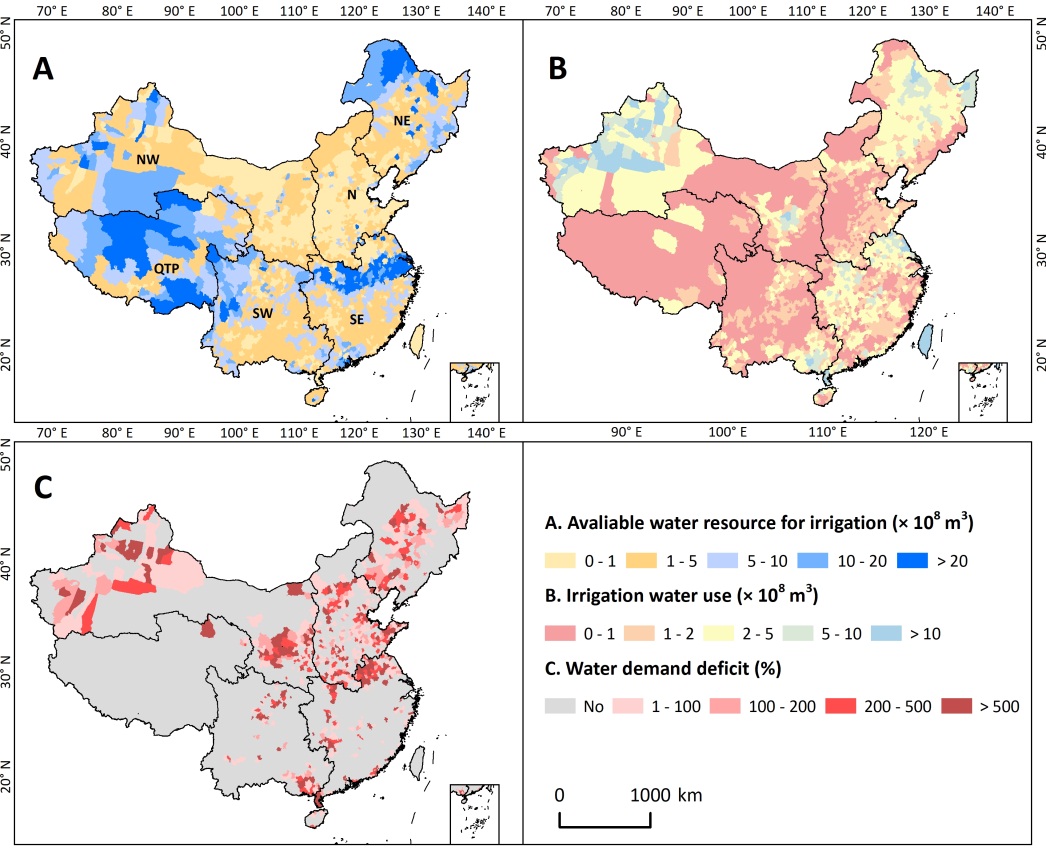


**Figure S5. Available water resources (A), irrigation water use (B), and water demand deficit ratio (C).** Note: NE, northeast China; NW, northwest China; N, north China; SE, southeast China; SW, southwest China; QTP, Qinghai-Tibet Plateau.

**
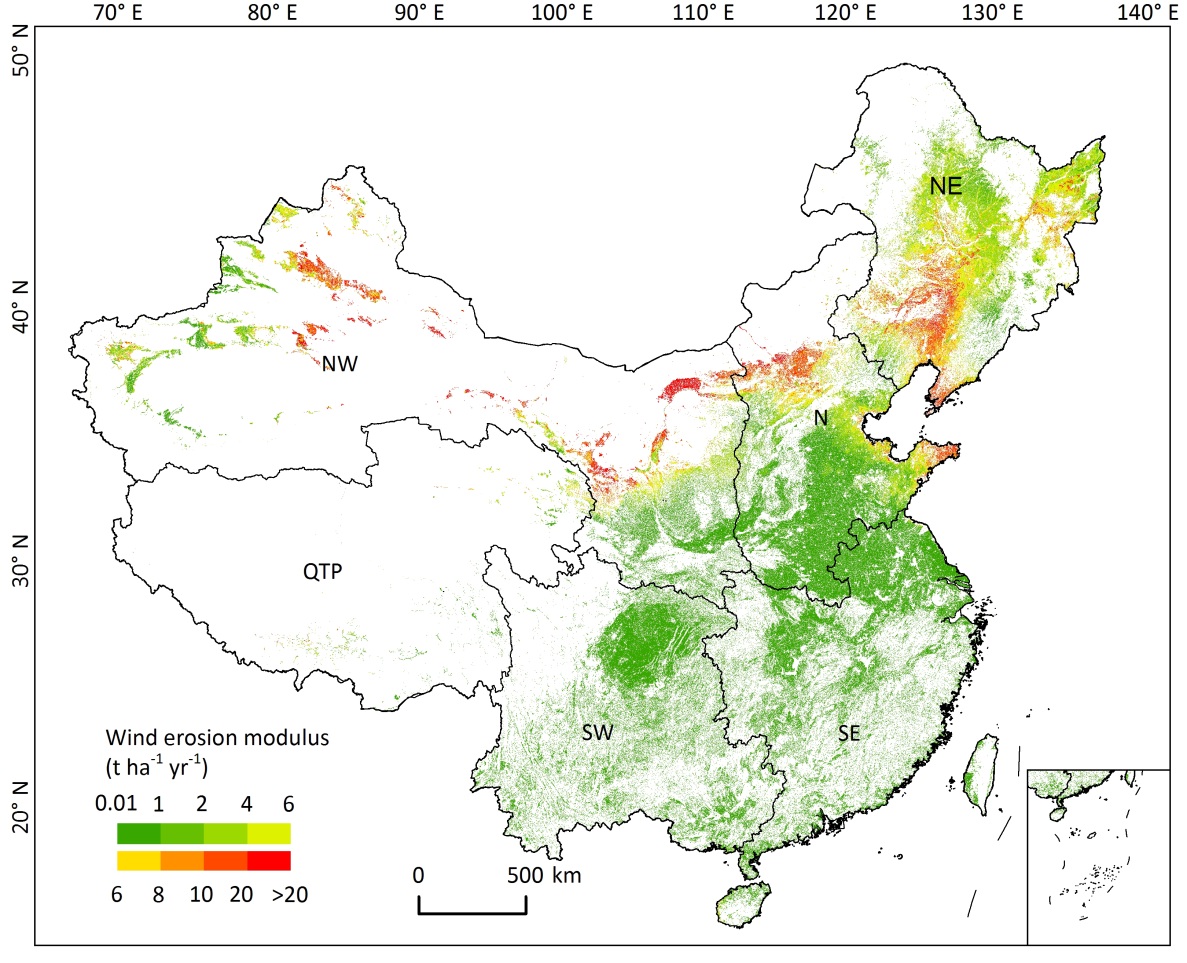
**

**Figure S6. Average wind erosion modulus for cropland between 1991 and 2015 at grid level (1 km x 1 km).** Note: NE, northeast China; NW, northwest China; N, north China; SE, southeast China; SW, southwest China; QTP, Qinghai-Tibet Plateau.

**
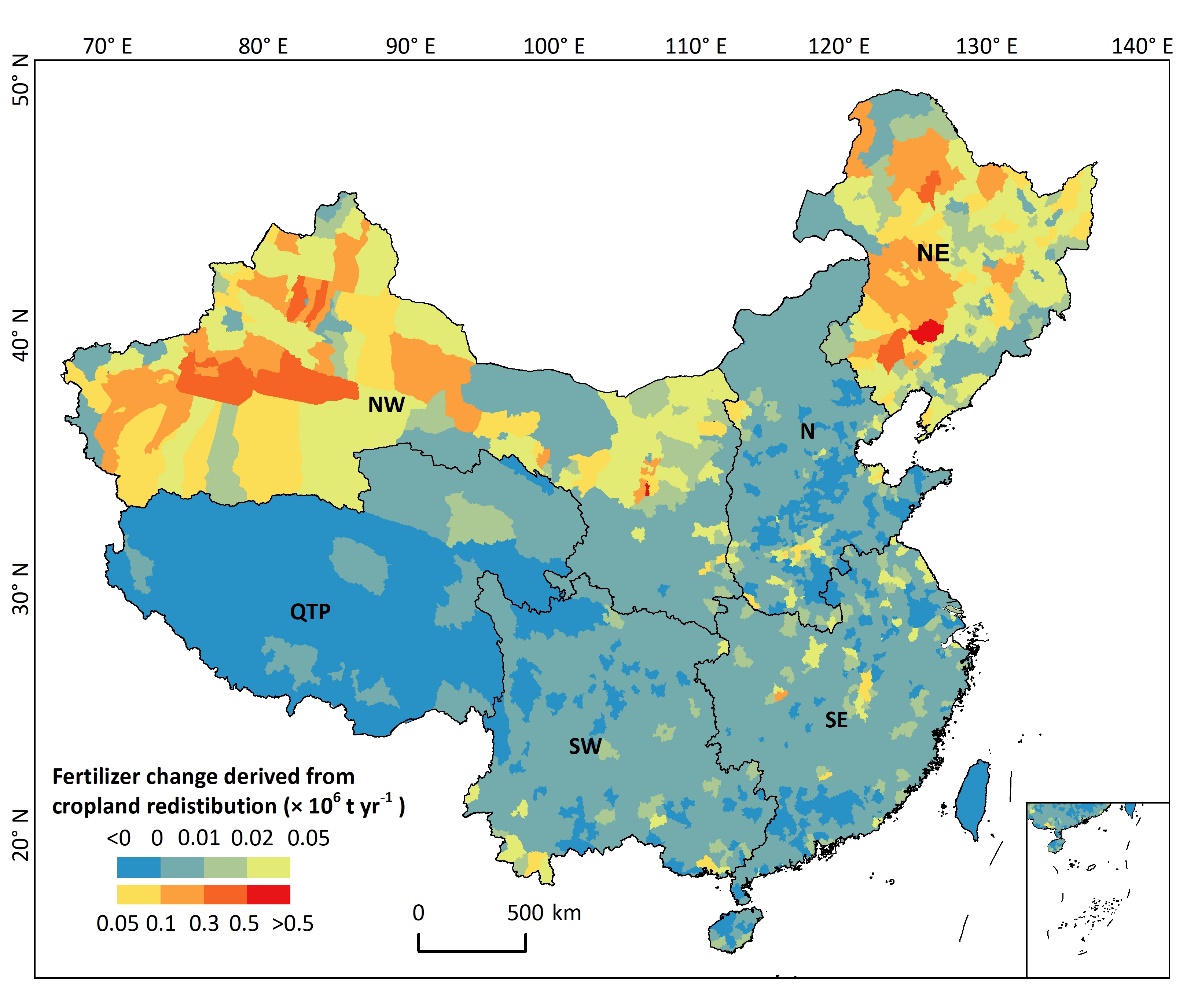
**

**Figure S7. The change in fertilizer use induced by cropland redistribution at county level.** Note: NE, northeast China; NW, northwest China; N, north China; SE, southeast China; SW, southwest China; QTP, Qinghai-Tibet Plateau.


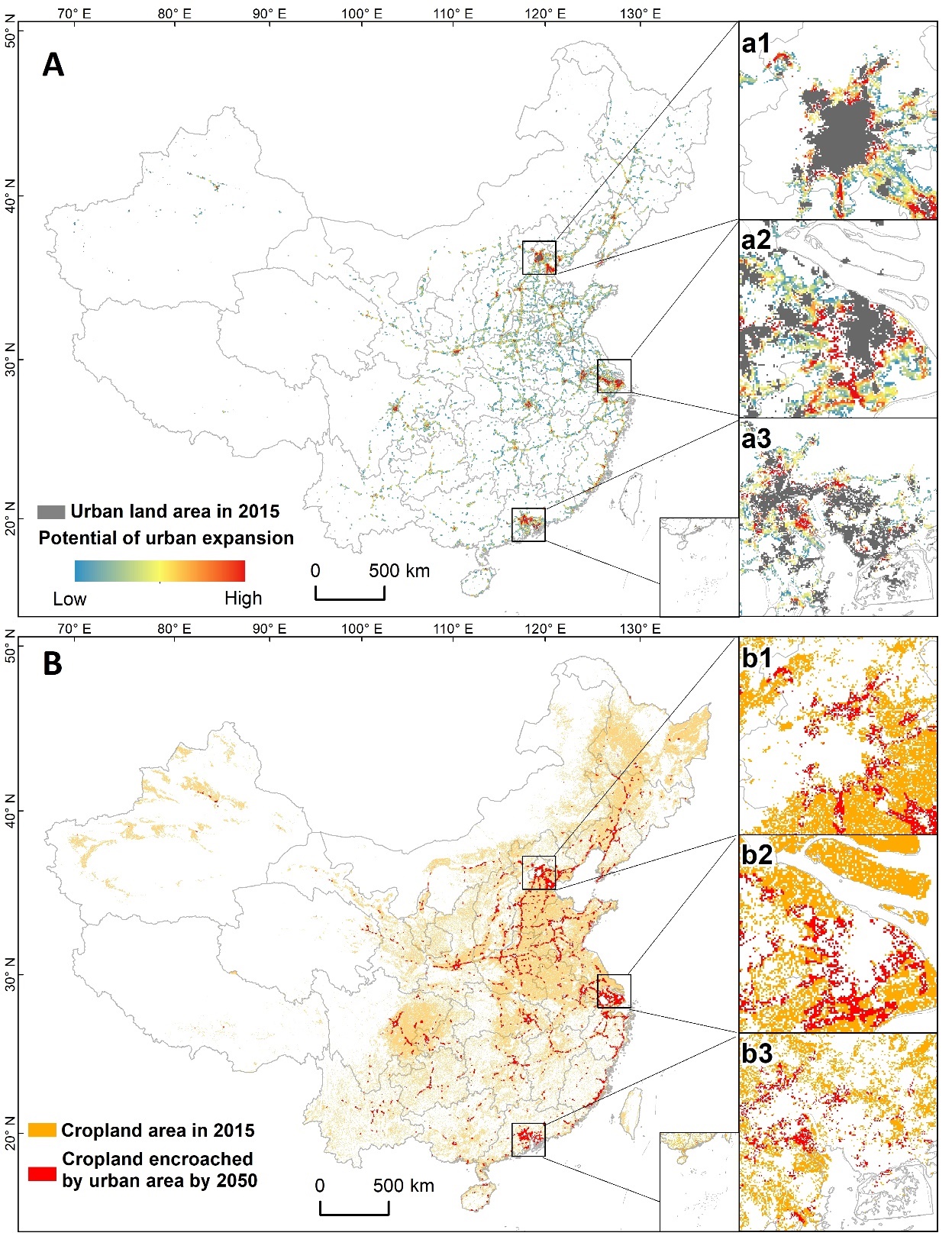


**Figure S8. Future urban expansion potential at grid level (1 km×1 km) (A) and the cropland encroachment from urbanization by 2050 (B).**

**
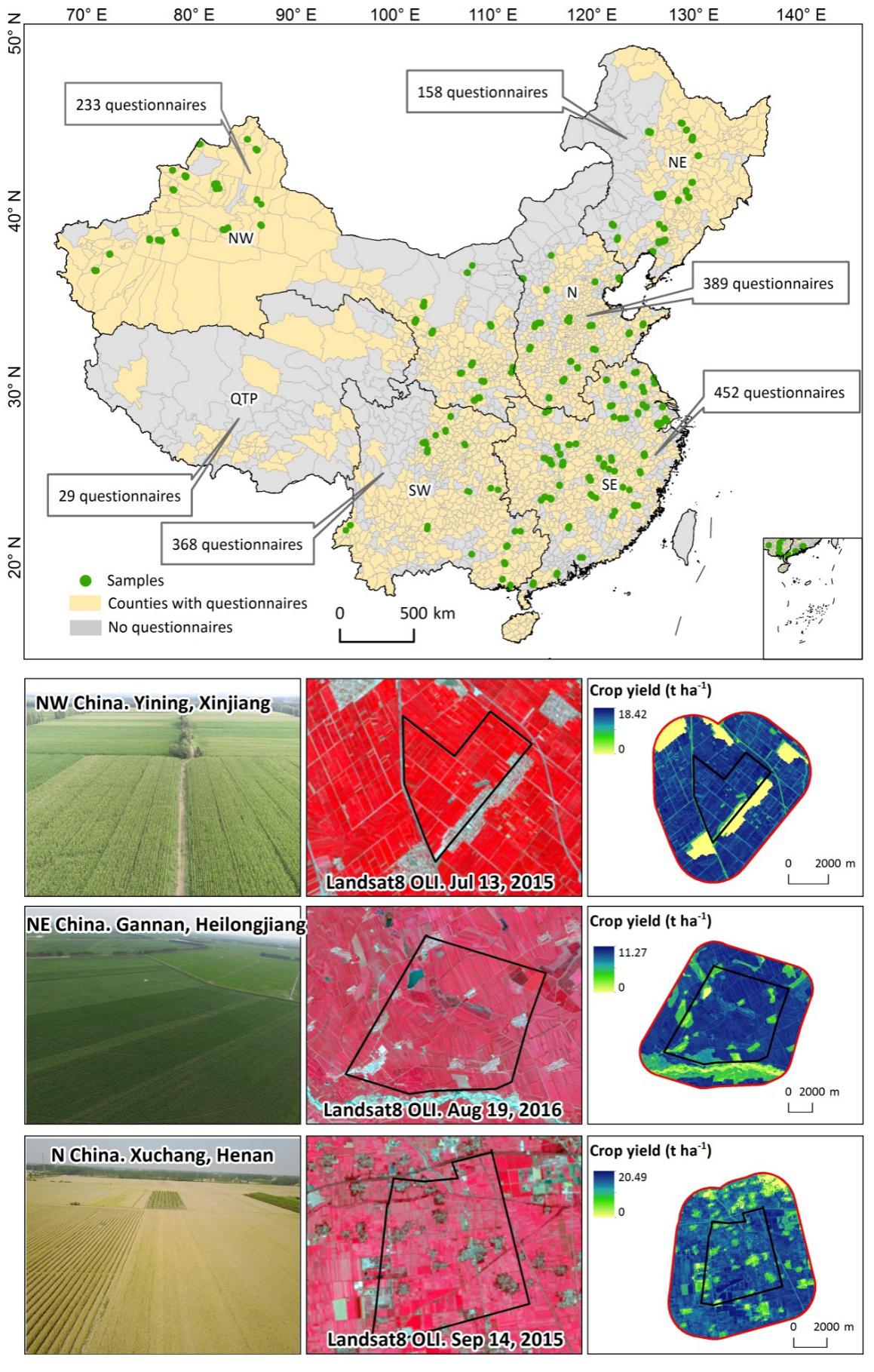
**

**Figure S9. Spatial distribution of sample locations for questionnaires.**


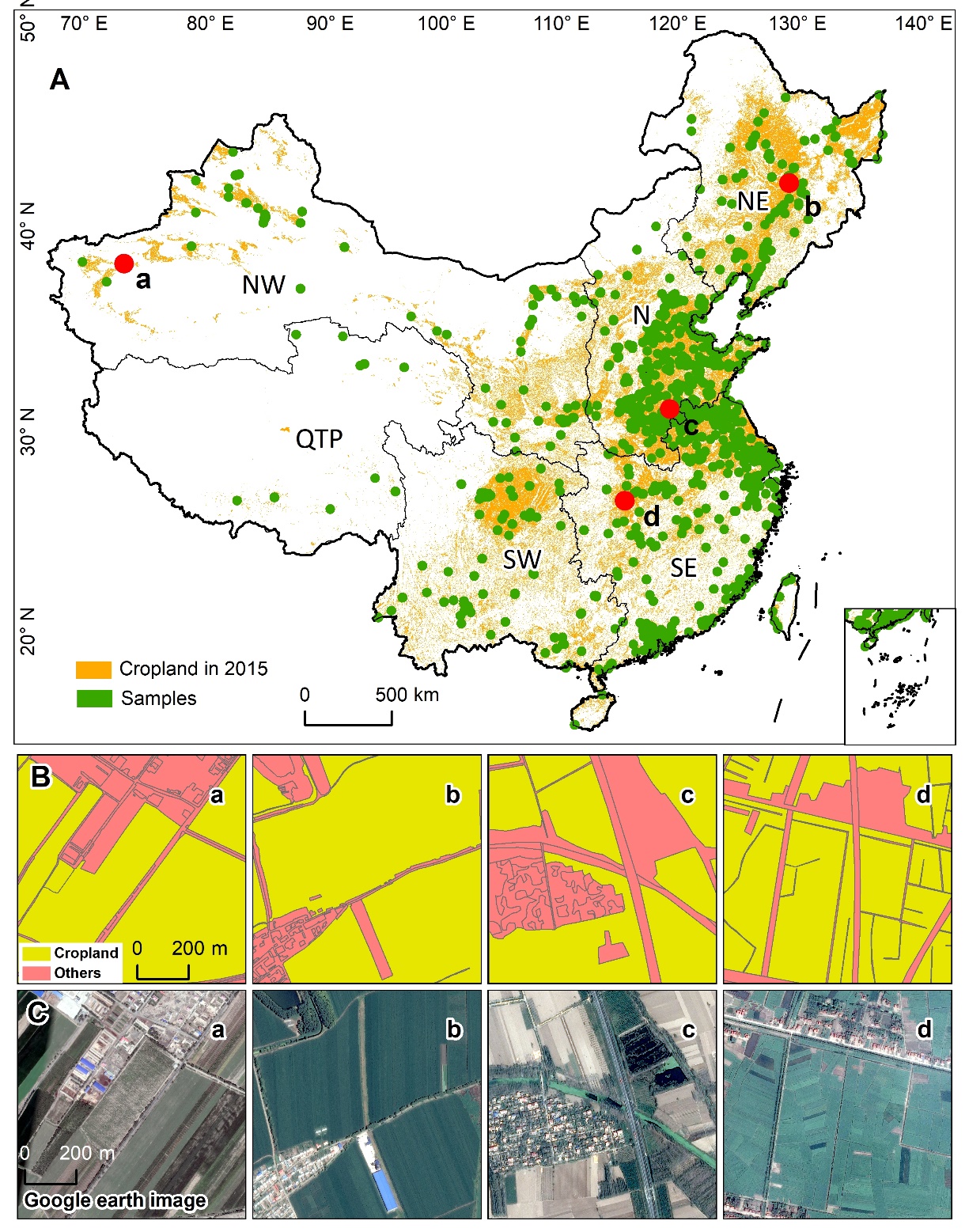


**Figure S10. Validation of cropland data.** (A) Validation samples for cropland of CLUD; (B) Cropland and other land-use types based on high-resolution images of sites a, b, c, d; (C) High-resolution images of sites a, b, c, d from Google Earth. Note: NE, northeast China; NW, northwest China; N, north China; SE, southeast China; SW, southwest China; QTP, Qinghai-Tibet Plateau.

**
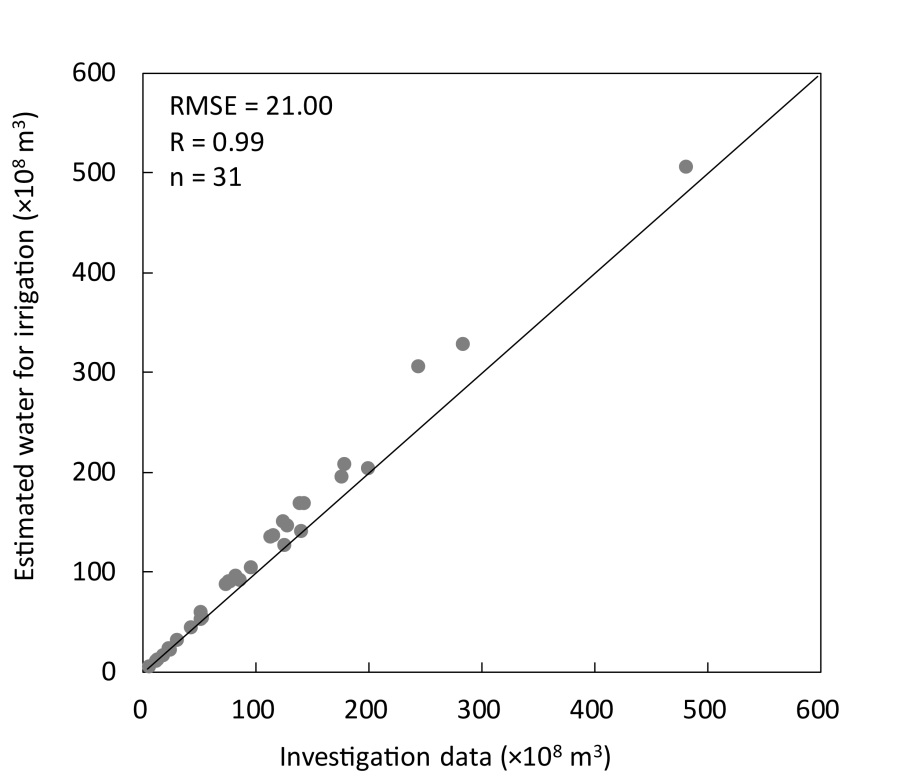
**

**Figure S11. Validation of irrigation water use dataset.**

**Table S1. Description of agro–big data.**

| Items | Description | Sources |
| --- | --- | --- |
| Cropland dynamics | Cropland patches at 5-year intervals, 1990–2015 derived from 30 mｘ30 m resolution images | China Land Use/cover Change Dataset (CLUD) (Supplementary Fig. S1) |
| Crop yields across China at county scale | The cereal yield of maize, rice, wheat, and others from each county from 1991 to 2015 | Annals of agricultural statistics from Ministry of Agriculture and Rural Affairs of the People’s Republic of China |
| Average grain productivity | Average cereal yield with 1 kmｘ1 km resolution from 2011 to 2015 | Remote sensing and statistical data  (Supplementary Fig. S3) |
| Irrigation water use | Available water resource of each county, irrigation water use, and water demand deficit in 1990–2015 | Ministry of Water Resources of the People’s Republic of China and questionnaires  (Supplementary Fig. S5) |
| Wind erosion | Average wind erosion modulus with 1 kmｘ1 km resolution during 1991–2015 | Revised wind erosion equation model (RWEQ) and ^137^Cs observation (Supplementary Fig. S6) |
| Cropland quality levels in 2015 | Fifteen levels of cropland quality were merged as excellent, good, moderate, and poor | Ministry of Land and Resources of the People’s Republic of China (Supplementary Fig. S2) |
| Questionnaires (1,629) and surveys (236) | Investigation on cropland use, resource consumption, environmental impacts in each county, and sample site surveys | State Agriculture Comprehensive Development Office of the People’s Republic of China (Supplementary, Fig. S9) |
| Cropland policies | State land-use and cultivated protection policies | Ministry of Land and Resources of the People’s Republic of China (Supplementary, Table S2) |

**Table S2. State policies for cropland management.**

| Policy | Year | Department | Main content |
| --- | --- | --- | --- |
| Prime farmland protection regulation | 1994 | State Council | The croplands located in prime farmland protection zones shall not be changed to another type of property without approval of the State Council. |
| Dynamic equilibrium of gross cropland | 1997 | State Council | Cropland loss should be replaced with equivalent quantity and quality of cropland to achieve dynamic equilibrium of the total yield of cropland. Farmland reduction, reclamation of cultivated land should be equivalent to the quantum and quality of the primary farmland occupied. |
| Cropland requisition- compensation balance | 1998 | National People’s Congress | The replacement land must be of equal grade or higher than the occupied cropland to maintain the total amount of cropland. In the case of cultivated land occupation, the same amount of land should be reclaimed. |
| “Grain for green” program | 2000 | State Council | State implemented the strategy and funding support for returning farmland to forests or grassland to improve ecosystem services in western China. |
| Rigorous land management policy | 2004 | State Council | State implemented strictest land-management policies to protect cropland, including law enforcement strengthening, preventing cropland occupation, responsibility system construction, et al. |

**Table S3. Cropland area statistics and dynamics at national and zonal levels, 1990–2015.**

| Zone | | Cropland area (×10^6^ ha) | | | | | |
| --- | --- | --- | --- | --- | --- | --- | --- |
|  |  | 1990 | 1995 | 2000 | 2005 | 2010 | 2015 |
| NE | | 30.67 | 33.22 | 34.64 | 34.94 | 35.03 | 35.14 |
| NW | | 19.36 | 19.53 | 19.97 | 20.39 | 20.98 | 21.85 |
| N | | 35.14 | 34.81 | 34.84 | 34.42 | 34.02 | 33.69 |
| SE | | 28.14 | 27.41 | 27.24 | 26.42 | 25.70 | 24.90 |
| SW | | 23.32 | 23.24 | 23.28 | 23.10 | 22.76 | 22.45 |
| QTP | | 1.42 | 1.42 | 1.46 | 1.46 | 1.46 | 1.45 |
| All of China | | 138.04 | 139.64 | 141.41 | 140.72 | 139.95 | 139.47 |
| Zone | Change | Cropland change (×10^6^ ha) | | | | | |
|  |  | 1990–1995 | 1995–2000 | 2000–2005 | 2005–2010 | 2010–2015 | 1990–2015 |
| NE | Gain | 3.19 | 1.78 | 0.57 | 0.26 | 0.23 | 6.04 |
|  | Loss | 0.63 | 0.37 | 0.27 | 0.17 | 0.12 | 1.56 |
|  | Net change | 2.56 | 1.41 | 0.30 | 0.09 | 0.11 | 4.47 |
| NW | Gain | 1.00 | 0.85 | 1.02 | 0.79 | 1.19 | 4.85 |
|  | Loss | 0.83 | 0.41 | 0.60 | 0.20 | 0.32 | 2.36 |
|  | Net change | 0.17 | 0.43 | 0.42 | 0.59 | 0.86 | 2.48 |
| N | Gain | 0.33 | 0.23 | 0.18 | 0.05 | 0.01 | 0.80 |
|  | Loss | 0.66 | 0.21 | 0.60 | 0.45 | 0.34 | 2.26 |
|  | Net change | -0.33 | 0.02 | -0.42 | -0.40 | -0.33 | -1.46 |
| SE | Gain | 0.11 | 0.13 | 0.10 | 0.06 | 0.11 | 0.50 |
|  | Loss | 0.84 | 0.30 | 0.92 | 0.77 | 0.91 | 3.74 |
|  | Net change | -0.73 | -0.17 | -0.82 | -0.72 | -0.80 | -3.24 |
| SW | Gain | 0.14 | 0.12 | 0.09 | 0.02 | 0.01 | 0.38 |
|  | Loss | 0.21 | 0.08 | 0.27 | 0.36 | 0.32 | 1.25 |
|  | Net change | -0.07 | 0.04 | -0.18 | -0.34 | -0.32 | -0.87 |
| QTP | Gain | 0.00 | 0.05 | 0.01 | 0.01 | 0.00 | 0.07 |
|  | Loss | 0.00 | 0.01 | 0.01 | 0.00 | 0.01 | 0.04 |
|  | Net change | 0.00 | 0.04 | 0.00 | 0.00 | -0.01 | 0.03 |
| All of China | Gain | 4.77 | 3.15 | 1.98 | 1.19 | 1.55 | 12.64 |
|  | Loss | 3.17 | 1.38 | 2.67 | 1.95 | 2.04 | 11.21 |
|  | Net change | 1.60 | 1.77 | -0.69 | -0.77 | -0.49 | 1.43 |

Note: NE, northeast China; NW, northwest China; N, north China; SE, southeast China; SW, southwest China; QTP, Qinghai-Tibet Plateau.

**Table S4. The geographical zones of China.**

| Zones | Covered provinces | Land area (×10^6^ ha) | Percentage of area (%) | Agricultural population (×10^6^) | Agricultural GDP (×10^9^ $) | Mean precipitation (mm yr^-1^) |
| --- | --- | --- | --- | --- | --- | --- |
| NE | Northeastern inner Mongolia, Jilin, Heilongjiang, Liaoning provinces | 125.02 | 13.15 | 72.19 | 68.27 | 531 |
| NW | Xinjiang, Gansu, West of inner Mongolia, Ningxia, Shanxi provinces | 280.92 | 29.56 | 45.19 | 70.99 | 215 |
| N | Central inner Mongolia, Beijing, Tianjin, Hebei, Henan, Shandong, Shaanxi provinces | 81.89 | 8.62 | 138.13 | 219.21 | 529 |
| SE | Jiangsu, Anhui, Shanghai, Hubei, Hunan, Jiangxi, Zhejiang, Fujian, Guangdong, Guangxi, Hunan, Hainan, Hong Kong, Macau, Taiwan provinces | 153.78 | 16.18 | 188.31 | 255.61 | 1489 |
| SW | Chongqing, Sichuan, Yunnan, Guizhou provinces | 113.86 | 11.98 | 85.25 | 145.77 | 1118 |
| QTP | Qinghai, Tibet provinces | 194.90 | 20.51 | 3.06 | 7.52 | 411 |
| Note: NE, northeast China; NW, northwest China; N, north China; SE, southeast China; SW, southwest China; QTP, Qinghai-Tibet Plateau. | | | | | | |

**Table S5. Effects of cropland change on grain production at national and zonal levels, 1990–2015.**

| Zone | | Change | Grain production change as a result of cropland gain or loss (×10^6^ t) | | | | | |
| --- | --- | --- | --- | --- | --- | --- | --- | --- |
|  |  |  | 1990–1995 | 1995–2000 | 2000–2005 | 2005–2010 | 2010–2015 | 1990–2015 |
| NE | Gain | | 10.41 | 5.87 | 2.12 | 0.93 | 0.79 | 20.11 |
|  | Loss | | 1.83 | 1.69 | 1.10 | 0.81 | 0.73 | 6.16 |
|  | Net change | | 8.57 | 4.18 | 1.02 | 0.12 | 0.05 | 13.94 |
|  | Percentage of national (%) | | 1.50 | 0.73 | 0.18 | 0.02 | 0.01 | 2.44 |
| NW | Gain | | 2.40 | 2.02 | 2.20 | 1.46 | 2.33 | 10.42 |
|  | Loss | | 2.73 | 1.38 | 1.38 | 0.62 | 1.17 | 7.28 |
|  | Net change | | -0.32 | 0.64 | 0.82 | 0.84 | 1.16 | 3.14 |
|  | Percentage of national (%) | | -0.06 | 0.11 | 0.14 | 0.15 | 0.20 | 0.55 |
| N | Gain | | 1.34 | 0.61 | 0.70 | 0.26 | 0.04 | 2.95 |
|  | Loss | | 4.02 | 1.07 | 3.31 | 2.64 | 1.92 | 12.96 |
|  | Net change | | -2.67 | -0.46 | -2.61 | -2.38 | -1.88 | -10.01 |
|  | Percentage of national (%) | | -0.47 | -0.08 | -0.46 | -0.42 | -0.33 | -1.75 |
| SE | Gain | | 0.90 | 0.99 | 1.09 | 0.66 | 0.94 | 4.58 |
|  | Loss | | 6.71 | 2.53 | 7.67 | 6.71 | 8.31 | 31.92 |
|  | Net change | | -5.81 | -1.54 | -6.58 | -6.05 | -7.37 | -27.34 |
|  | Percentage of national (%) | | -1.01 | -0.27 | -1.15 | -1.06 | -1.29 | -4.78 |
| SW | Gain | | 0.52 | 0.46 | 0.30 | 0.07 | 0.04 | 1.40 |
|  | Loss | | 1.41 | 0.44 | 1.20 | 2.00 | 1.72 | 6.77 |
|  | Net change | | -0.89 | 0.02 | -0.89 | -1.93 | -1.67 | -5.36 |
|  | Percentage of national (%) | | -0.15 | 0.00 | -0.16 | -0.34 | -0.29 | -0.94 |
| QTP | Gain | | 0.00 | 0.03 | 0.01 | 0.01 | 0.00 | 0.06 |
|  | Loss | | 0.01 | 0.02 | 0.04 | 0.01 | 0.04 | 0.11 |
|  | Net change | | 0.00 | 0.01 | -0.03 | -0.01 | -0.03 | -0.06 |
|  | Percentage of national (%) | | 0.00 | 0.00 | -0.01 | 0.00 | -0.01 | -0.01 |
| All of China | Gain | | 15.58 | 9.98 | 6.42 | 3.39 | 4.15 | 39.51 |
|  | Loss | | 16.70 | 7.13 | 14.69 | 12.79 | 13.89 | 65.20 |
|  | Net change | | -1.12 | 2.85 | -8.28 | -9.40 | -9.74 | -25.69 |
|  | Percentage of national (%) | | -0.20 | 0.50 | -1.45 | -1.64 | -1.70 | -4.49 |

Note: NE, northeast China; NW, northwest China; N, north China; SE, southeast China; SW, southwest China; QTP, Qinghai-Tibet Plateau.

**Table S6. Effects of cropland change on irrigation water use and water demand deficit.**

| Zone | Available water resource  (×10^8^ m^3^) | | | | Irrigation water use in 1990 (×10^8^ m^3^) | | Irrigation water use in 2015 (×10^8^ m^3^) | | | | Irrigation water change caused by cropland change in 1990-2015  (×10^8^ m^3^ yr^-1^) | | | | Irrigation water change caused by cropland gain in 1990-2015 (×10^8^ m^3^ yr^-1^) | | | |  |
| --- | --- | --- | --- | --- | --- | --- | --- | --- | --- | --- | --- | --- | --- | --- | --- | --- | --- | --- | --- |
| NE | 1358.61 | | | | 532.57 | | 597.06 | | | | 64.49 | | | | 100.40 | | | |  |
| NW | 1108.44 | | | | 568.68 | | 734.75 | | | | 166.07 | | | | 278.65 | | | |  |
| N | 714.71 | | | | 530.10 | | 510.11 | | | | -19.99 | | | | 11.62 | | | |  |
| SE | 9152.79 | | | | 1665.59 | | 1452.63 | | | | -212.96 | | | | 40.88 | | | |  |
| SW | 2093.90 | | | | 544.41 | | 533.72 | | | | -10.69 | | | | 10.59 | | | |  |
| QTP | 1049.06 | | | | 39.82 | | 40.39 | | | | 0.57 | | | | 1.54 | | | |  |
| All of China | 15477.51 | | | | 3881.17 | | 3868.66 | | | | -12.51 | | | | 443.68 | | | |  |
| Water demand deficit | 1%–100% | | 100%–200% | | | | | | 200%–500% | | | >500% | | | | Total | | | |
|  | Area  (×10^6^ ha) | Proportion  (%) | | Area  (×10^6^ ha) | | Proportion  (%) | | Area  (×10^6^ ha) | | Proportion  (%) | | | Area  (×10^6^ ha) | Proportion  (%) | | | Area  (×10^6^ ha) | Proportion  (%) |  |
| NE | 1.67 | 27.64 | | 0.53 | | 8.78 | | 0.66 | | 11.00 | | | 0.32 | 5.35 | | | 6.04 | 52.77 |  |
| NW | 0.85 | 17.46 | | 0.62 | | 12.78 | | 0.28 | | 5.87 | | | 0.93 | 19.26 | | | 4.85 | 55.38 |  |
| N | 0.19 | 23.42 | | 0.04 | | 4.72 | | 0.10 | | 12.68 | | | 0.06 | 7.49 | | | 0.80 | 48.31 |  |
| SE | 0.05 | 10.88 | | 0.01 | | 2.34 | | 0.02 | | 3.31 | | | 0.04 | 8.84 | | | 0.50 | 25.37 |  |
| SW | 0.01 | 2.94 | | 0.00 | | 1.00 | | 0.04 | | 9.50 | | | 0.00 | 1.13 | | | 0.38 | 14.57 |  |
| QTP | 0.00 | 0.89 | | 0.00 | | 0.54 | | 0.00 | | 0.30 | | | 0.01 | 9.39 | | | 0.07 | 11.12 |  |
| All of China | 2.77 | - | | 1.20 | | - | | 1.10 | | - | | | 1.37 | - | | | 12.64 | - |  |

Note: NE, northeast China; NW, northwest China; N, north China; SE, southeast China; SW, southwest China; QTP, Qinghai-Tibet Plateau.

**Table S7. Effects of cropland change and “Grain for green” program on wind erosion.**

|  | | | Change in wind erosion (×10^6^ t yr^-1^) | | | | | |
| --- | --- | --- | --- | --- | --- | --- | --- | --- |
| Zone | | Types | 1990–1995 | 1995–2000 | 2000–2005 | 2005–2010 | 2010–2015 | 1990–2015 |
| NE | Cropland gain | | 2.01 | 0.56 | 0.19 | 0.16 | 0.18 | 3.11 |
|  | “Grain for green” | | 0.14 | -0.21 | -0.09 | 0.00 | 0.00 | -0.16 |
| NW | Cropland gain | | 1.80 | 2.94 | -0.32 | 1.67 | 1.55 | 7.64 |
|  | “Grain for green” | | -2.97 | -1.50 | -0.54 | -0.34 | -0.89 | -6.24 |
| N | Cropland gain | | 0.22 | 0.13 | 0.14 | -0.04 | 0.02 | 0.47 |
|  | “Grain for green” | | -0.07 | -0.11 | -0.07 | -0.04 | -0.03 | -0.32 |
| SE | - | | - | - | - | - | - | - |
|  | - | | - | - | - | - | - | - |
| SW | - | | - | - | - | - | - | - |
|  | - | | - | - | - | - | - | - |
| QTP | Cropland gain | | 0.01 | 0.06 | 0.00 | 0.01 | 0.00 | 0.08 |
|  | “Grain for green” | | 0.00 | 0.00 | -0.01 | 0.00 | 0.00 | -0.01 |
| All of China | Cropland gain | | 4.04 | 3.69 | 0.01 | 1.81 | 1.75 | 11.29 |
|  | “Grain for green” | | -2.91 | -1.82 | -0.72 | -0.39 | -0.91 | -6.75 |

Note: NE, northeast China; NW, northwest China; N, north China; SE, southeast China; SW, southwest China; QTP, Qinghai-Tibet Plateau.

**Table S8. Effects of cropland reclamation on fertilizer use at national and zonal levels, 1990–2015.**

| Zone | | Fertilizer use increase as a result of cropland gain (×10^6^ t yr^-1^) | | | | | | | | | | | |
| --- | --- | --- | --- | --- | --- | --- | --- | --- | --- | --- | --- | --- | --- |
|  |  | 1990–1995 | | 1995–2000 | | 2000–2005 | | 2005–2010 | | 2010–2015 | | 1990–2015 | |
| NE | 0.58 | | 0.31 | | 0.13 | | 0.04 | | 0.04 | | 1.10 | |  |
| NW | 0.25 | | 0.22 | | 0.25 | | 0.20 | | 0.30 | | 1.22 | |  |
| N | 0.13 | | 0.04 | | 0.05 | | 0.02 | | 0.00 | | 0.24 | |  |
| SE | 0.07 | | 0.08 | | 0.05 | | 0.03 | | 0.07 | | 0.30 | |  |
| SW | 0.06 | | 0.04 | | 0.03 | | 0.01 | | 0.00 | | 0.14 | |  |
| QTP | 0.00 | | 0.01 | | 0.00 | | 0.00 | | 0.00 | | 0.01 | |  |
| All of China | 1.09 | | 0.70 | | 0.51 | | 0.30 | | 0.41 | | 3.01 | |  |

Note: NE, northeast China; NW, northwest China; N, north China; SE, southeast China; SW, southwest China; QTP, Qinghai-Tibet Plateau.

**Table S9. Projections of total population, urbanization rate, and cropland area in China, 2020–2050.**

| Year | Total population (×10^8^) | | | Urbanization rate (%) | Cropland area (×10^6^ ha) | | |
| --- | --- | --- | --- | --- | --- | --- | --- |
| 1990 | 11.43 | | | 26.41 | 138.04 | | |
| 1995 | 12.11 | | | 29.04 | 139.64 | | |
| 2000 | 12.67 | | | 36.22 | 141.41 | | |
| 2005 | 13.08 | | | 42.99 | 140.72 | | |
| 2010 | 13.41 | | | 49.95 | 139.95 | | |
| **2015** | **13.75** | | | **56.10** | **139.47** | | |
| Future | Low scenario | Medium scenario | High scenario | Predicted | Low scenario | Medium scenario | High scenario |
| 2020 | 14.43 | 14.62 | 14.81 | 61.52 | 138.34 | 138.40 | 138.46 |
| 2025 | 14.64 | 15.01 | 15.37 | 65.88 | 137.48 | 137.61 | 137.75 |
| **2030** | **14.72** | **15.29** | **15.86** | **69.23** | **136.79** | **137.00** | **137.22** |
| 2035 | 14.52 | 15.21 | 15.91 | 71.60 | 136.40 | 136.67 | 136.94 |
| 2040 | 14.16 | 15.05 | 15.95 | 73.25 | 136.13 | 136.49 | 136.85 |
| 2045 | 13.72 | 14.82 | 15.95 | 74.80 | 135.89 | 136.35 | 136.80 |
| **2050** | **13.18** | **14.52** | **15.94** | **76.30** | **135.66** | **136.25** | **136.81** |

**Table S10. Projected grain demand and supply, wind erosion, and irrigation water use changes caused by cropland change in China, 2020–2050.**

| Year | Grain demand (×10^6^ t) | | | Grain production (×10^6^ t) | | | Irrigation water use increase  (×10^8^ m^3^ yr^-1^) | | | Wind erosion increase  (×10^6^ t yr^-1^) | | | Fertilizer increase (×10^6^ t yr^-1^) | | |
| --- | --- | --- | --- | --- | --- | --- | --- | --- | --- | --- | --- | --- | --- | --- | --- |
| 1990 | 457.33 | | | 407.81 | | | 0.00 | | | 0.00 | | | 0.00 | | |
| 1995 | 484.48 | | | 416.11 | | | 140.82 | | | 4.04 | | | 1.09 | | |
| 2000 | 506.97 | | | 405.22 | | | 185.63 | | | 7.73 | | | 1.79 | | |
| 2005 | 523.02 | | | 427.76 | | | 288.10 | | | 7.74 | | | 2.30 | | |
| 2010 | 536.36 | | | 496.37 | | | 362.74 | | | 9.55 | | | 2.60 | | |
| **2015** | **549.85** | | | **572.28** | | | **443.68** | | | **11.29** | | | **3.01** | | |
| Future | Low scenario | Medium scenario | High scenario | Low scenario | Medium scenario | High scenario | Low scenario | Medium scenario | High scenario | Low scenario | Medium scenario | High scenario | Low scenario | Medium scenario | High scenario |
| 2020 | 625.49 | 633.66 | 641.84 | 567.62 | 567.89 | 568.15 | 510.92 | 515.19 | 519.46 | 12.45 | 12.53 | 12.6 | 3.33 | 3.35 | 3.37 |
| 2025 | 683.21 | 700.23 | 717.26 | 564.13 | 564.66 | 565.21 | 558.84 | 567.76 | 576.43 | 13.28 | 13.44 | 13.59 | 3.55 | 3.59 | 3.63 |
| **2030** | **736.18** | **764.50** | **792.81** | **561.27** | **562.16** | **563.04** | **594.06** | **608.49** | **622.92** | **13.9** | **14.14** | **14.39** | **3.72** | **3.78** | **3.85** |
| 2035 | 726.01 | 760.66 | 795.33 | 559.68 | 560.81 | 561.92 | 612.38 | 630.44 | 648.77 | 14.21 | 14.53 | 14.84 | 3.80 | 3.89 | 3.97 |
| 2040 | 708.22 | 752.65 | 797.44 | 558.56 | 560.05 | 561.51 | 619.02 | 642.85 | 666.96 | 14.33 | 14.74 | 15.16 | 3.83 | 3.94 | 4.06 |
| 2045 | 686.00 | 741.09 | 797.74 | 557.58 | 559.47 | 561.32 | 622.07 | 652.15 | 683.06 | 14.38 | 14.9 | 15.44 | 3.85 | 3.99 | 4.13 |
| **2050** | **659.21** | **726.14** | **796.99** | **556.64** | **559.07** | **561.37** | **621.36** | **658.75** | **698.36** | **14.37** | **15.02** | **15.7** | **3.84** | **4.02** | **4.20** |

**Table S11. Classification system of China Land Use/cover Dataset.**

| First-level class | Second-level  class | Definition |
| --- | --- | --- |
| Cropland | - | Cultivated land for crops, including mature cultivation, new cultivation, fallow, and shifting cultivation; intercropping land such as crop-fruit, crop-mulberry, and crop-forest in which a crop is a dominant species that has been cultivated for at least three years. |
|  | Paddyland | Cropland that has enough water supply and irrigation facilities for planting paddy rice, lotus, etc., including rotation land for paddy rice and dry farming crops. |
|  | Dryland | Cropland for cultivation without water supply and irrigating facilities; cropland for dry farming crops that has water supply and irrigation facilities; cropland for planting vegetables; fallow land. |
| Woodland | | Land for growing trees, including arbour, shrub, bamboo, and for forestry use. |
| Grassland | | Land covered by herbaceous plants with coverage greater than 5%, including shrub rangeland and mixed rangeland with shrub canopy coverage less than 10%. |
| Water body | | Land covered by natural water bodies or land with facilities for irrigation and water reservation. |
| Built-up land | | Land used for urban and rural settlements, factories, and transportation systems. |
| Unused land | | Land that is not put into practical use or is difficult to use, including sandy land, Gobi, Salina, bare soil, etc. |

**Table S12. Validation of national cropland data from CLUD.**

**12.1. Accuracy assessment for the developed cropland datasets.**

| Year | Accuracy for cropland | | | | Accuracy of first-level land classification | | Source |
| --- | --- | --- | --- | --- | --- | --- | --- |
|  | Samples size | PA (%) | UA (%) | OA (%) | Samples | Accuracy (%) |  |
| 1990 | 22,710 |  |  | 97.33 | 44,381 | 97.25 | Liu et al. 2003 [52] |
| 1995 | 46,828 |  |  | 99.22 | 46,828 | 98.72 | Liu et al. 2005 [53]; Zhang et al. 2014 [55] |
| 2000 | 99,867 |  |  | 99.09 | 33,929 | 98.04 | Zhang et al. 2014 [55] |
| 2005 | 11,701 |  |  | 99.30 | 35,877 | 98.56 | Zhang et al. 2014 [55] |
| 2010 | 7,875 | 91.98 | 92.16 |  | 7,875 | 89.86 | Kuang et al. 2016 [28] |
| 2015 | 2,200 | 90.36 | 93.03 |  | 2,200 | 91.95 | This study |

Note: PA, producer’s accuracy; UA, user’s accuracy; OA, overall accuracy

**12.2. Confusion matrix of land-use classification in 2015.**

| Land type | Ground truth (GT) samples (pixels) | | | | | | Total | User’s accuracy (%) |
| --- | --- | --- | --- | --- | --- | --- | --- | --- |
|  | Cropland | Woodland | Grassland | Water body | Built-up land | Unused land |  |  |
| Cropland | 347 | 9 | 6 | 1 | 5 | 5 | 373 | 93.03 |
| Woodland | 15 | 433 | 12 | 3 | 5 | 8 | 476 | 90.97 |
| Grassland | 9 | 19 | 508 | 0 | 3 | 26 | 565 | 89.91 |
| Water body | 2 | 0 | 0 | 48 | 0 | 1 | 51 | 94.12 |
| Built-up land | 7 | 3 | 0 | 0 | 237 | 1 | 248 | 95.56 |
| Other | 4 | 6 | 23 | 1 | 3 | 450 | 487 | 92.40 |
| Total | 384 | 470 | 549 | 53 | 253 | 491 | 2,200 | / |
| PA (%) | 90.36 | 92.13 | 92.53 | 90.57 | 93.68 | 91.65 | Overall accuracy = 91.95% | |

**Supplementary References**

Liu JY, Zhang ZX and Zhuang DF *et al.* A study on the spatial-temporal dynamic changes of land-use and driving forces analyses of China in the 1990s. *Geogr Res* 2003; **22**: 1–12.

Liu JY, Zhang ZX and Zhuang DF *et al*. *Remote Sensing Information Study of Land Use Change in China in 1990s*. Beijing: Science Press, 2005.

Zhang ZX, Wang X and Zhao XL *et al*. A 2010 update of National Land Use/Cover Database of China at 1:100000 scale using medium spatial resolution satellite images. *Remote Sens Environ* 2014; **149**: 142–154.

Kuang WH, Liu JY and Dong JW *et al*. The rapid and massive urban and industrial land expansions in China between 1990 and 2010: A CLUD-based analysis of their trajectories, patterns, and drivers. *Landsc Urban Plan* 2016; **145**: 21–33.
